# Canopy reflectance as a predictor of soil microbial community composition and diversity at a continental scale

**DOI:** 10.1111/nph.70720

**Published:** 2025-11-19

**Authors:** Angela Harris, Richard D. Bardgett

**Affiliations:** ^1^ Department of Geography, School of Environment Education and Development (SEED) University of Manchester Oxford Road Manchester M13 9PL UK; ^2^ Lancaster Environment Centre Lancaster University Lancaster LA1 4YQ UK

**Keywords:** canopy reflectance, foliar traits, imaging spectroscopy, NEON, soil microbial community

## Abstract

- Canopy reflectance captures plant traits related to ecological processes, which may reflect the composition of soil microbial communities. However, the extent to which canopy reflectance can help elucidate soil microbial community composition and diversity across biomes remains unclear.
- Using data from 14 National Ecological Observatory Network ecoregions (domains), we linked plant traits to soil microbial composition and diversity (characterised by phospholipid fatty acids and 16S rRNA gene sequencing) and built partial least squares regression models to predict soil microbial attributes from airborne imaging spectroscopy at the continental scale.
- The ability of remote sensing to predict soil microbial communities was mediated by plant attributes that both directly influence microbial communities and reflect shared responses to soil and climate gradients. Model validation accuracy varied with taxonomic resolution (normalised root mean squared error, 10.1–24%; coefficient of determination, 0.27–0.86), with models of broad soil microbial groups performing best, although bacterial community composition and diversity could also be modelled with moderate levels of accuracy (normalised root mean squared error, 12.5–18.6%; coefficient of determination, 0.43–0.61).
- Models using full‐spectrum hyperspectral data consistently outperformed those based on simple vegetation indices, highlighting the value of imaging spectroscopy for soil microbial research.

## Introduction

Soil microbial communities are fundamental to biological diversity and play a pivotal role in maintaining ecosystem functioning and health (Delgado‐Baquerizo *et al*., 2018a). Understanding the distribution of these communities at regional and global scales is essential for gaining insights into how soil microbial communities vary across ecosystems and environmental gradients and how they may influence or respond to large‐scale shifts in vegetation, climate or land use (Wallenstein & Hall, 2012). However, the identification of soil microbial communities across large spatial scales through *in situ* sampling presents significant logistical challenges and is often prohibitively expensive. Attempts to quantitatively predict soil microbial communities in unobserved locations are frequently hindered by an incomplete understanding of the factors governing the spatial and temporal patterns in their composition, diversity and functioning (Bardgett & van der Putten, 2014; Averill *et al*., 2021). At continental and global scales, climate, land use and soil abiotic properties such as organic carbon, pH and nutrient content, shape the composition and diversity of soil microbial communities (Tedersoo *et al*., 2014; Delgado‐Baquerizo *et al*., 2018b; Bickel & Or, 2020). However, evidence also points to the importance of plant community composition and functional diversity (i.e. the type and distribution of plant functional traits) as important determinants of soil microbial communities over large spatial extents (10^2^–10^3^ km) where differences in plant traits between species or functional groups are often most pronounced (Wardle & Zackrisson, 2005; de Vries *et al*., 2012; Delgado‐Baquerizo *et al*., 2018a; Crowther *et al*., 2019).

Empirical and theoretical evidence suggest that plant communities dominated by acquisitive traits, such as high specific leaf area (SLA) and elevated leaf nitrogen content, are associated with higher rates of photosynthesis and growth, more fertile soils with a higher abundance of bacteria relative to fungi, accelerated decomposition and faster nutrient cycling (Wardle *et al*., 2004; de Vries *et al*., 2012; Grigulis *et al*., 2013). Conversely, in plant communities characterised by more conservative traits, such as low SLA, nitrogen‐poor leaves and lower rates of growth and photosynthesis, soil microbial communities are dominated by fungi, including mycorrhizal fungi, relative to bacteria and rates of decomposition and nutrient cycling are slower (Wardle *et al*., 2004; de Vries *et al*., 2012; Grigulis *et al*., 2013). Yet, many field‐based studies have focused on specific ecosystems (predominantly grasslands), and the nature and significance of the reported aboveground to belowground associations are not always consistent (Grigulis *et al*., 2013; Porazinska *et al*., 2018; Buzzard *et al*., 2019). The extent to which the aboveground plant community characteristics are predictably associated with changes in soil microbial communities across large spatial extents and diverse land cover types, therefore, requires further investigation.

Large‐scale geographic predictions lend themselves well to the use of remotely sensed data. A plant's reflectance signature encompasses numerous foliar chemical, structural and physiological traits crucial for resource acquisition and stress tolerance. Yet, whilst many studies have utilised canopy reflectance to assess aboveground biodiversity or elucidate ecosystem functions (Kokaly *et al*., 2009; Williams *et al*., 2021; Zheng *et al*., 2023), and some have attempted to link plant spectral reflectance to soil abiotic properties such as moisture and pH (Dehaan & Taylor, 2002; Uno *et al*., 2005), fewer studies have used the notion of plant–soil microbe associations to link plant spectra to soil microbial communities. Those that have either used plant canopy reflectance to characterise the overlying habitat or environmental niche (Madritch *et al*., 2020; Lin *et al*., 2021; Sousa *et al*., 2021; Skidmore *et al*., 2022, 2025; Yu *et al*., 2024), or to predict ecosystem productivity and foliar traits that are thought to be associated with the soil microbiome (Cavender‐Bares *et al*., 2022). Both approaches rely on the presence of close associations between aboveground and belowground systems, although few remote sensing studies explicitly include *in situ* measurements of both plant and soil microbial attributes when linking soil microbial communities with spectral reflectance and those that do have focused on specific ecosystems such as forests or grasslands (Madritch *et al*., 2020; Cavender‐Bares *et al*., 2022). There remains a gap in our understanding as to the likely drivers of model performance across a wider range of ecosystems and thus where and when such models could be applied more broadly.

Our primary goal was to determine the potential of imaging spectroscopy to predict soil microbial community composition and diversity across a broad range of ecosystem types at the continental scale. Using open‐source continental‐scale data collected by the National Ecological Observatory Network (NEON), we first used field‐measured observations to determine the extent to which foliar traits are associated with microbial community composition and diversity. We subsequently used airborne imaging spectroscopy to predict microbial composition and diversity directly from canopy spectral reflectance and used model coefficients together with field‐measured data to interpret the spectrally based models. We tested our ability to model broad‐scale patterns in soil microbial biomass and key functional groups characterised by phospholipid fatty acids (PLFAs), together with bacterial taxonomic microbial composition and diversity measured using 16s rRNA gene sequencing, to provide a comprehensive understanding of the extent to which spectral reflectance may hold potential for predicting soil microbial communities and their diversity.

Specifically, our objectives were: (1) to determine whether aboveground foliar trait variation is linked to soil microbial community composition and diversity at the continental scale and (2) to determine whether soil microbial community composition and diversity can be predicted across multiple ecosystems at the continental scale using canopy spectral reflectance, and if so, to determine which microbial community attributes are likely to be predicted most accurately.

We expected strong relationships between measured foliar traits associated with resource acquisition and conservation strategies and the composition and diversity of soil microbial communities, given the substantial structural and phytochemical differences among plant communities at the continental scale, which both influence or are influenced by soil properties. Furthermore, because plant canopy spectral reflectance is sensitive to both structural and chemical attributes of the canopy, we also anticipated that canopy spectra would be indirectly related to soil microbial communities through associations with plant attributes and soil conditions. Additionally, we hypothesised that broad soil microbial community and functional groups (e.g. characterised by PLFAs) would be better predicted by canopy reflectance than finer‐scale taxonomic estimates of community composition and diversity (e.g. based on 16S sequencing), consistent with patterns observed in other studies and among macro‐organisms (Averill *et al*., 2021).

## Materials and Methods

### Spatial and temporal sampling design

NEON constitutes a continental‐scale ecological observatory infrastructure employing standardised protocols for robust spatiotemporal data collection across North America (Keller *et al*., 2008). NEON's sampling strategy follows a nested design structured across four spatial levels: domains, sites, plots and cores, ranging from broad regional coverage to fine‐scale local measurements. The United States, including Alaska, Hawaii and Puerto Rico, is divided into 20 ecoclimatic domains using multivariate geographic clustering based on nine environmental variables, including seasonal precipitation and solar insolation (Hargrove & Hoffman, 2004). Each domain contains one permanent core site and up to two additional sites that are designed to move location every 5–10 yr. There are a total of 47 terrestrial sites (5–215 km^2^) located within the 20 NEON domains (Kao *et al*., 2012; Thorpe *et al*., 2016). Each terrestrial site encompasses up to 34 base plots (40 m × 40 m) that contain a central 20 m × 20 m area that is designated for co‐located measurements including plant foliar chemistry. Soil samples are collected from randomly selected locations within the remaining 10 m outer margin of each plot (Meier *et al*., 2023). Foliar traits are collected every 5 yr, soil biochemical sampling occurs annually and soil microbial sampling occurs annually at core sites and every 5 yr at other sites. NEON's Airborne Observation Platform (AOP) uses a small aircraft outfitted with remote sensing equipment (including an imaging spectrometer) to fly over sites annually, weather permitting.

### Plot selection based on near‐synchronous observations

For our analyses, we obtained information on soil microbial communities, soil biochemical properties, site‐level climate, selected foliar traits and hyperspectral airborne data from NEON's open access data portals (Supporting Information Table S1). At the time of data acquisition, NEON had only been operating for a relatively short period. Since the datasets we used followed differing sampling schedules, there was limited spatial and temporal overlap among some of the datasets, meaning that not all variables were collected for each plot simultaneously. Consequently, we developed a targeted data selection strategy and applied filtering criteria aimed at maximising both the number and geographic coverage of plots where soil microbial and airborne data were jointly available, while also minimising the time lag between their respective sampling events. Specifically, we obtained near‐synchronous soil microbial community composition and airborne data by first obtaining all available airborne imagery and microbial data collected between 2014 and 2021, that is, from the start of NEON's soil microbial sampling campaign to the most recent data available at the time of access. These data were subsequently filtered to retain only plots where soil microbial data were available within ±60 d of an airborne image. The ±60‐d threshold was chosen through trial and error as a balance between minimising temporal discrepancies and retaining a sufficient number of plots for analysis. We further restricted the dataset to natural land covers by removing plots classified by NEON as cultivated land and only selected plots sampled during NEON‐defined peak greenness periods, excluding those collected during seasonal transitions. We used the timing and location of these core near‐synchronous observations as a reference point for filtering all other datasets used in the analyses (i.e. soil properties, climate and foliar traits) to maximise temporal alignment. Plots were included if, in addition to microbial data, either soil properties or foliar trait data were also available (long‐term climate data were available for all plots as they were collected at the site level). Consequently, all data were aligned with the airborne imagery within a ±60‐d window and with each other within ±120 d. The largest temporal mismatches occurred between the foliar trait and soil datasets, which resulted in eight plots where foliar data were collected between 61 and 70 d before or after the corresponding soil sampling event. We pooled data from all available years to maximise data availability. However, where a plot was sampled in multiple years, we retained only the year with the smallest time gap between the airborne and soil microbial datasets. Further details on additional filtering, such as quality control exclusions, handling of missing variables and managing uncertainty in aggregating soil core and leaf‐level measurements to the plot scale, as well as any data sub‐setting that was required for particular statistical analyses, are provided in the subsequent methodological subsections.

**Fig. 1:**
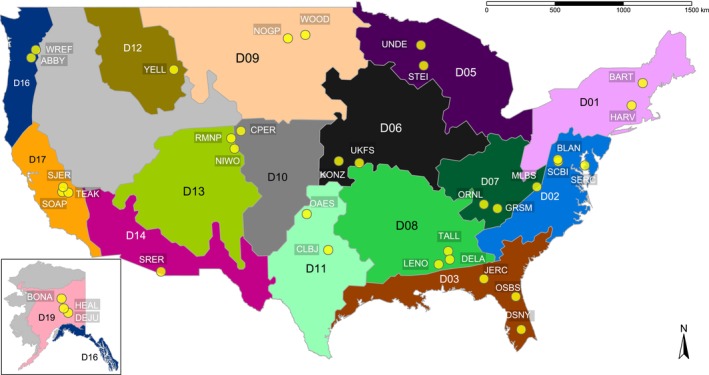
Locations of the 34 sites (yellow dots) sampled over 16 National Ecological Observatory Network ecoclimatic domains (highlighted and labelled) across the continental United States and Alaska. The site and domain code names are listed in Supporting Information Table S2.

**Fig. 2:**
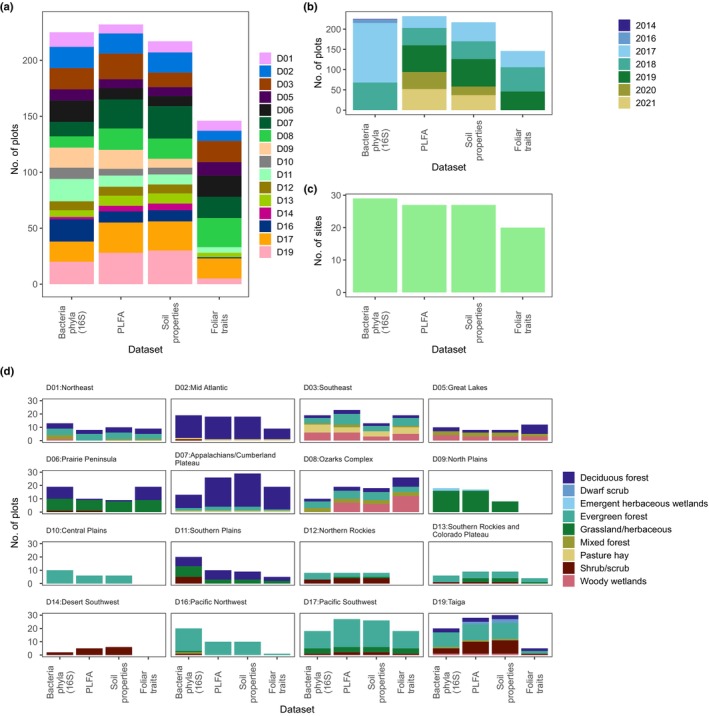
A summary of the datasets used including (a) the number of plots located within each National Ecological Observatory Network (NEON) ecoclimatic domain; (b) the number of plots per year; (c) the number of NEON sites within which the plots were located and (d) the distribution of land cover classes within each dataset.

After all filtering criteria had been applied, the final dataset contained plots from 34 terrestrial NEON sites, which spanned 16 ecoclimatic domains within the NEON network and included diverse habitat types such as forests, grasslands and wetlands (Figs 1, 2; Table S2). A total of 232 plots were available for modelling soil microbial community composition from spectral reflectance based on PLFAs, and 255 plots were available for modelling soil bacterial composition and diversity from 16S rRNA gene sequencing (Fig. 2).

### Microbial biomass and coarse‐level functional groups

To enable direct comparison between soil biogeochemical and microbial datasets, all analyses are conducted on the same material whenever possible. Details regarding sampling, storage and processing of all soils used in the analyses can be found in Methods S1 and references therein.

We used the NEON soil microbe biomass data product (DP1.10104.001; Table S1) to obtain quantitative proxy estimates of broad soil microbial groups measured by PLFA. In brief, soil samples collected in the field were kept moist before analyses at a domain laboratory, where they were homogenised to minimise spatial heterogeneity within each horizon and coarse debris was removed. A chloroform‐methanol extraction method was used to extract microbial lipid biomarkers from a representative subsample, which were subsequently analysed against a reference standard for quantification using either gas chromatography mass spectrometry (before 2018) or gas chromatography (from 2019 onwards). Following correction for extraction efficiency, we summed the concentrations of i14:0, i15:0, i16:0, i17:0, a15:0 and a17:0 for Gram‐positive bacteria, cyclo17:0 and cyclo19:0 for Gram‐negative bacteria; 10Me16:0, 10Me17:0, 10Me18:0 for Actinomycetales (Gram‐positive); 16:1ω7 *cis*, 18:2ω6, 18:3ω6 for saprophytic fungi (SF) and 16:1ω5 for arbuscular mycorrhizal (AM) fungi. We also calculated the total amount of PLFAs as a measure of the total viable microbial biomass (Frostegård *et al*., 1991); the ratio of fungal to bacterial PLFAs (F : B), which is commonly used as an indicator of shifts in the relative abundance of fungi and bacteria and their relative roles in nutrient cycling and channelling of energy through the soil food web (Bardgett & McAlister, 1999; de Vries *et al*., 2013; Chomel *et al*., 2022); and finally the ratio of Gram‐positive to Gram‐negative bacteria PLFAs (G+ : G−) as an indicator of the relative carbon availability for soil bacterial communities in organic soils, where a higher ratio can indicate a shift towards more stable and complex carbon sources in the soil and a lower ratio can indicate a reliance on simpler, more labile carbon compounds (Fanin *et al*., 2019). All measured lipids were present in each of the samples, and summed concentrations (nmol g) and ratios of PLFAs were calculated for individual soil cores before aggregating from the core to the plot (see details in Methods S2). Further details regarding NEON's soil microbe biomass processing chain and quality assurance can be found in Stanish (2025).

### Bacterial community composition and diversity

We complemented the PLFA with 16S rRNA gene sequencing to provide information regarding the members of the bacterial community that are present, their relative abundance and their diversity. Sequencing data were obtained from the NEON soil microbe community composition data product (DP1.10081.001; Table S1). Briefly, after field collection, samples were frozen on dry ice and transported to ultra‐low freezers at the NEON field laboratories and subsequently shipped to an analytical laboratory where DNA extraction, sample library preparation and DNA sequencing occurred (Methods S1). Sequencing data were quality filtered and processed using the DADA2 pipeline and dereplicated into Amplicon sequence variants (ASVs) before being assigned to taxonomic groups using the SILVA database (https://www.arbsilva.de/fileadmin/silva_databases/qiime/Silva_132_release.zip). Complete information on sample processing, DNA extraction, sequencing library preparation and DNA sequencing can be found in Stanish (2025b). We subsequently removed any undefined taxa and those assigned as Archaea before rarefying the observations to 5000 reads per sample (mean = 16 258, SD = 10 911) and removed samples with fewer than 5000 reads from the analysis.

We measured bacterial community composition at the phylum and individual sequencing levels (i.e. ASV) given that previous studies have indicated that predictability may depend on taxonomic scale (Averill *et al*., 2021). At the level of phylum, we modelled the dominant phyla defined as those that were present across all plots and represented the top 10% of phyla when ranked by relative abundance (i.e. rRNA reads). We summed all other phyla within each sample to generate the category ‘Other’. The relative abundance per phylum (per sample) was calculated by dividing the raw reads of each phylum by the total number of reads in the sample. We also characterised bacterial community composition based on an abundance matrix of ASVs and applied non‐metric multidimensional scaling (NMDS) analysis using Bray–Curtis dissimilarities (vegan package; Oksanen *et al*., 2022) to represent gradients of bacterial community composition in low‐dimensional space (e.g. Griffiths *et al*., 2011; Cavender‐Bares *et al*., 2022). Finally, we calculated bacterial richness (the number of ASVs) as an indicator of bacterial diversity (Delgado‐Baquerizo *et al*., 2018a).

### Soil properties, foliar traits and climate data

We used the NEON soil physical and chemical properties product (DP1.10086.001; Table S1) to obtain data on soil pH, organic carbon and nitrogen content, the carbon‐to‐nitrogen ratio (C : N) and soil moisture (SM) for samples with horizon depths of 30 cm or less. We obtained information on 17 plant physical and chemical foliar traits from the NEON foliar traits product (DP1.10026.001; Table S1), which included pigments, carbon, nitrogen, key elements, cellulose and lignin. Full details of all the traits measured can be found in Table S3, and further details regarding foliar sampling are provided in Methods S3. We also obtained site‐level information on long‐term mean annual temperature (MAT) and mean annual precipitation (MAP) from the NEON field site documentation available from https://www.neonscience.org/field‐sites/explore‐field‐sites (Table S3).

To facilitate integration of field data with airborne imagery and to aid comparisons between field datasets collected across different spatial extents (e.g. soil cores vs individual plants), we employed a spatial scaling approach using Bayesian models to estimate plot‐level Bayesian average values for foliar traits, soil properties, soil microbial biomass, soil community composition at the phylum level and measures of bacterial richness (Averill *et al*., 2021, see details in Methods S2). Plot‐level community matrices of ASVs were obtained by simple averaging across the cores within each plot, due to the high number of ASVs present across the dataset (> 270 000).

### Processing NEON Airborne Observation Platform (AOP) imagery

The NEON spectrometer orthorectified surface direction reflectance mosaic product (DP3.30006.001; Table S1) was used to obtain plot‐level spectral data for model development and validation. The hyperspectral instrument collects visible to shortwave infrared data with a spatial resolution of 1 metre. The Full Width at Half Maximum for each band is *c*. 5 nm, with a total of 426 bands spanning from 380 to 2500 nm. Before mosaicking, each flight line is orthorectified and atmospherically corrected to surface reflectance using ATCOR‐4 (Richter & Schläpfer, 2002; Schläpfer & Richter, 2002). The central wavelengths for each spectral band are slightly different for image data collected in different years due to annual sensor calibration; thus, we resampled all spectra to a common wavelength range using the year 2019. For each selected plot, we generated a 20 m diameter circular buffer around the centre of the plot (i.e. the area designated for non‐destructive sampling) and averaged the spectra within the buffer to obtain one mean reflectance signature per plot. We removed noisy and atmospheric absorption bands and retained spectral data spanning 403–1334, 1450–1785 and 1971–2396 nm ranges. We also removed plots with low vegetation coverage by deleting plots with a Normalised Difference Vegetation Index (NDVI) value < 0.4, calculated as (*R*_804_ − *R*_673_)/(*R*_804_ + *R*_673_), where *R*_804_ and *R*_673_ are the reflectance at 804 and 673 nm, respectively. We vector‐normalised the retained spectra by dividing the reflectance at every wavelength by the full‐spectrum reflectance norm so that the effects of internal canopy shade were minimised (Feilhauer *et al*., 2010; Wang *et al*., 2020). The range of vector‐normalised reflectance for each domain and land cover type is illustrated in Figs S1 and S2.

### Statistical analyses and modelling

Our primary goal was to determine the potential of imaging spectroscopy to quantify soil microbial community composition and diversity at the continental scale. Given that any links between reflectance and the soil microbial community would be indirect via plant canopies and their relationship with soil properties, we also investigated the strength and nature of associations between soil microbial community attributes and measured foliar traits, climate and soil abiotic properties.

We first conducted exploratory ordination analyses (principal component analysis (PCA) and NMDS) to observe how foliar traits, soil properties and soil microbial community composition and diversity varied across the study area. A centred log‐ratio transformation was applied to phylum‐level data before PCA to account for their compositional nature. We then used variation partitioning modelling to disentangle the relative contributions of foliar traits, land cover, soil properties, climate and location, in explaining variation in the soil microbiome (Table S3). To reduce collinearity within predictor groups, we performed PCA and used the leading axes (explaining > 80% of variance) as inputs for variation partitioning for each predictor group. To assess the statistical significance of the unique contributions of each variable group, we conducted permutation‐based tests using partial redundancy analysis (partial RDA). Specifically, for each group of predictors, a partial RDA was run while conditioning on the remaining groups. We used 999 permutations to assess significance. Phylum‐level relative abundance and ASV community data were Hellinger‐transformed before variation partitioning to reduce the influence of highly abundant taxa and to meet the assumptions of RDA. Since not all field variables were measured in conjunction with the soil microbial data, we used a subset of plots where all data were available (*n* = 96 and *n* = 53 for PLFA and 16S rRNA sequencing data, respectively).

We complemented our variation partitioning modelling with correlation analysis (Spearman's rank and partial correlations) to allow us to assess the direction and strength of associations between specific foliar traits and soil microbial communities (i.e. PLFA groups, bacterial phyla, richness and community composition). We selected pH, SM, MAT, MAP and C : N ratio as confounding variables for the partial correlations, as each was strongly correlated (rho < 0.7) with one or more of the other measured soil properties, but not with each other. Highly correlated variables were identified using the Caret package in R (Kuhn, 2008). The complete correlation matrices between individual variables are shown in Fig. S3.

To predict soil microbial community composition and diversity from spectral reflectance, we developed a series of partial least squares regression (PLSR; Haaland & Thomas, 1988) models. Separate models were constructed for each measure of the soil microbiome. PLSR has been widely used in developing spectral models where there is a multi‐collinearity issue in the predictor variables (i.e. spectra), and the number of predictors largely exceeds the number of observations (Serbin *et al*., 2014; Asner & Martin, 2016; Ely *et al*., 2019; Wang *et al*., 2019, 2020). Before model development, we followed the approach of Wang *et al*. (2020) to remove model outliers by running each PLSR model 200 times, calculating the mean absolute error between the measured and predicted values and subsequently removing 5% of data with the highest errors in each model. We split the resulting dataset into calibration (70%) and out‐of‐sample validation (30%) datasets. The out‐of‐sample validation dataset was not used at any point in model development. We used the calibration dataset in a jackknife data permutation approach to first determine the optimal number of PLSR model components for each model. Specifically, for each microbial community, we built 200 PLSR models using random subsets (70%) of the calibration dataset and validated each model using the remaining 30% of data. The prediction residual error sum of squares statistic was calculated for each component across all PLSR models and used to identify the optimal number of model components for each final SMC model (Burnett *et al*., 2021). We developed the final PLSR models by permuting the calibration data, randomly selecting 70% of the data each time to build 200 separate models per microbial community. Each of the 200 models was then applied to the withheld out‐of‐sample validation dataset to calculate the mean and SD of the model estimates, with the latter being used to estimate uncertainty of the model predictions for each soil microbial community. All model performances were evaluated using the coefficient of determination (*R* ^2^) between measured and predicted soil microbial community composition values, the root mean squared error of prediction (RMSE), the normalised RMSE (NRMSE = RMSE/range) and model bias. As a baseline to compare with the performance of the PLSR models, we also created separate linear models using just the NDVI or normalised difference water index (NDWI; Gao, 1996) spectral indices as an indicator of vegetation biomass/productivity and SM, respectively. We calculated NDWI as (*R*_860_ − *R*_1240_)/(*R*_860_ + *R*_1240_), where *R*_860_ and *R*_1240_ are the reflectance at 860 and 1240 nm, respectively.

All statistical analyses were undertaken in R. PCA was implemented using the package factominer (Lê *et al*., 2008), NMDS, variation partition modelling and RDA were implemented using the package vegan (Oksanen *et al*., 2022), and partial correlation analysis was implemented using ppcor (Kim, 2015). The PLSR modelling was implemented using the spectratrait package (Burnett *et al*., 2021).

## Results

### Continental‐scale patterns of foliar traits, soil properties and soil microbial communities

**Fig. 3:**
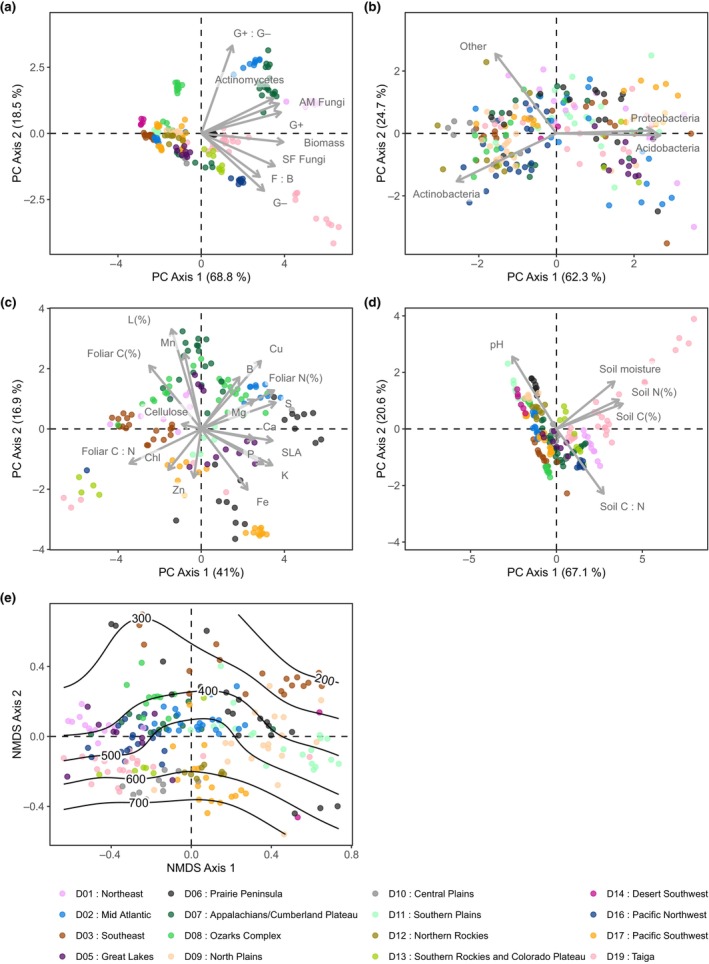
Ordinations (principal components (PCA) and non‐metric multidimensional scaling (NMDS)) of (a) phospholipid fatty acids (PLFA) groups (*n* = 232), (b) dominant bacterial phyla (*n* = 225), (c) foliar traits (*n* = 146), (d) soil properties (*n* = 217) and (e) bacterial community composition (amplicon sequence variants (ASVs); *n* = 255), with ASV richness shown as contour lines. Points are coloured according to the National Ecological Observatory Network domain.

Across the sampled locations, there was substantial variation in plant traits, soil properties and climate as well as in soil microbial communities (Figs S4–S7). Exploratory PCA analyses of the data revealed that many foliar traits, soil properties and PLFAs associated with key microbial groups were spatially clustered according to NEON domains rather than land cover class (Figs 3, S8). Continental‐scale variation in PLFA groups was primarily driven by overall microbial group abundance (PC1) and the ratio of Gram‐positive to Gram‐negative bacteria (PC2; Fig. 3a; Table S4). In comparison, variation in foliar traits broadly followed the leaf economic spectrum (Wright *et al*., 2004), with acquisitive traits such as nitrogen, SLA, sulphur and potassium loading positively on PC1, while high carbon‐to‐nitrogen ratios indicated more conservative strategies (Fig. 3c; Table S5). Variation in lignin concentration contributed to the separation of plots along foliar PC2. Soil properties also showed clear spatial clustering, with PC1 capturing gradients in carbon, nitrogen and moisture and PC2 reflecting a trade‐off between pH and the carbon‐to‐nitrogen ratio (Fig. 3d; Table S6). Bacterial community composition based on ASV data was similarly spatially structured along both NMDS axes, with NMDS axis 2 negatively associated with bacterial richness (i.e. number of ASVs; Fig. 3e). By contrast, the relative abundance of bacterial phyla did not exhibit clear spatial clustering by NEON domain (Fig. 3b). Variation along PC1 reflected opposing relative abundances of Acidobacteria, Actinobacteria and Proteobacteria, with Acidobacteria negatively correlated with relative abundances of both Actinobacteria and Proteobacteria. PC2 captured variation in the relative abundance of the ‘other’ less dominant bacterial phyla that were present within the plots (Fig. 3b; Table S7).

### The relative importance of foliar traits in explaining soil microbial community composition

**Fig. 4:**
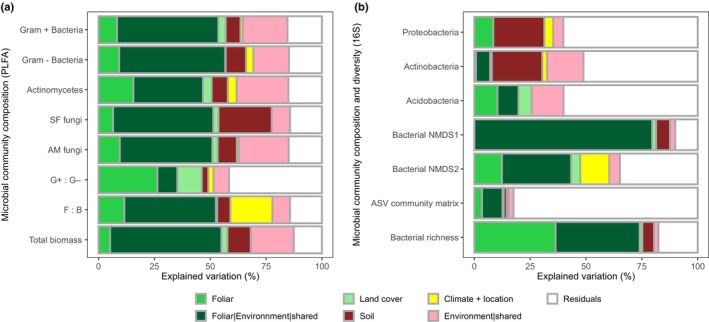
Contribution of foliar traits to microbial community composition and diversity across National Ecological Observatory Network (NEON) ecoclimatic domains. Partition modelling was used to evaluate the unique and shared portions of variation in (a) broad microbial groups (phospholipid fatty acids (PLFA)) and (b) bacterial phyla, the first two non‐metric multidimensional scaling (NMDS) axes of amplicon sequence variant (ASV) bacteria community matrix, the ASV community abundance matrix and bacterial richness, explained by foliar traits, land cover, soil properties and climate + location. Environment|shared refers to the percent of shared variation in microbial composition explained by land cover, soil and climate + location (i.e. excluding foliar traits). Foliar|Environment|shared refers to the percent of shared variation in microbial composition explained by foliar traits, land cover, soil and climate + location. Only complete datasets were used in partition modelling (PLFA *n* = 96; 16S bacterial communities *n* = 53). *P*‐values associated with the unique proportion of variation explained by different groups of predictors are available in Supporting Information Table S8.

Variation partitioning indicated that foliar traits were a significant explanatory factor underlying the variation in broad PLFA groups and ratios (Fig. 4a; Table S8). Foliar traits explained the greatest share of unique explained variation across the 8 PLFA soil microbial attributes, followed by soil, climate and location and land cover, respectively (41%, 33%, 14% and 12%, respectively). Furthermore, when considering both the unique variation explained by foliar traits and the variation they share with environmental factors (including land cover, soil properties and climate and location), foliar traits accounted for half of the total observed variation in the PLFA data across the NEON domains. Foliar traits were particularly good unique predictors of the PLFA biomarkers, indicating the G+ : G− ratio (26%), the abundance of Actinomycetes (16%) and the F : B ratio (12%; Fig. 4a).

Soil properties explained the greatest share of the unique explained variation in the relative abundance across all bacterial phyla (58%). In comparison, foliar traits accounted for 26%, while climate, location and land cover type each explained < 10%. Soil properties, rather than foliar traits, were more important in explaining variation in the relative abundance of both Proteobacteria and Actinobacteria, whereas the opposite was true for Acidobacteria (Fig. 4b; Table S8). Soil properties also explained the greatest proportion of unique variation in the first bacterial NMDS axis (6%) and foliar traits explained the most for the second axis (13%), although variation that was shared between foliar traits and all other environmental factors consistently contributed to most of the explained variation in both axes (78% and 31%; for axis 1 and 2, respectively; Fig. 4b; Table S8). Foliar traits explained a far higher proportion of unique variation in bacterial richness than soil properties (37% and 5%, respectively). Foliar traits were also better unique predictors than soil properties in explaining the variation in the more complex ASV bacterial community matrix (4% and 1%, respectively; Table S8). While all variation partitioning models were statistically significant (*P* < 0.001), the models of bacterial relative abundance and the ASV community composition matrix exhibited higher levels of unexplained variation.

**Fig. 5:**
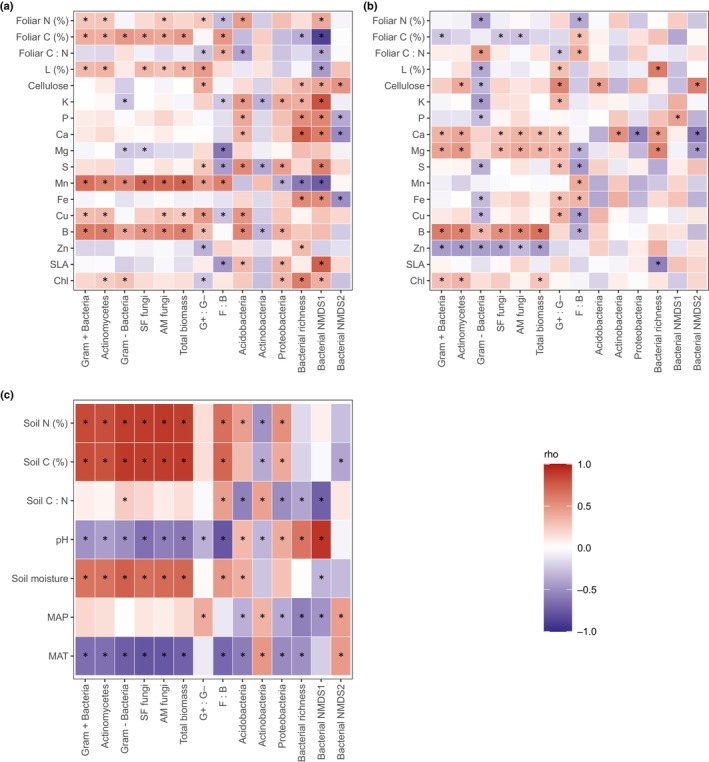
Correlations (Spearman) between soil microbial communities and (a) foliar traits, (b) foliar traits controlled for by soil and climate and (c) soil properties and climate attributes. Only complete datasets were used in the correlation analysis (phospholipid fatty acids, *n* = 96; bacteria (16S), *n* = 53). Stars indicate significant correlations (*P* < 0.05). A Bonferroni correction has been applied to all correlations (*P* < 0.05). AM, arbuscular mycorrhizal; SLA, specific leaf area.

Fig. 5 shows the results of Spearman and partial Spearman correlation analysis, which was used to complement variation partitioning by providing insight into the specific foliar traits most strongly associated with microbial composition and diversity, beyond what was captured by the multivariate models of grouped variables. We initially found significant associations between several foliar traits that can be linked to the plant economics spectrum and microbial PLFA group abundances and ratios (Fig. 5a). Significant trait associations were generally positive for all broad taxonomic groups and mirrored the patterns of association observed for soil carbon, nitrogen and moisture (Fig. 5c). Magnesium and potassium were exceptions, showing negative correlations with Gram‐negative bacteria PLFAs. Magnesium was also negatively correlated with SF. By contrast, both PLFA ratios and the ratio of fungi‐to‐bacteria in particular, showed more nuanced relationships with plant traits. For example higher relative levels of fungi were negatively associated with resource‐acquisitive traits (e.g. SLA, sulphur, magnesium, potassium and nitrogen) but positively correlated with foliar carbon. When the influence of soil and climate was reduced through partial correlations (Fig. 5b), several previously significant correlations became insignificant, indicating that some of the observed foliar trait associations were either driven by shared dependence with soil and/or climatic conditions or soil and/or climate variation had a similar effect on both the soil microbiome and foliar traits. Some foliar traits (e.g. boron) remained significantly correlated with the PLFA groups, or showed greater significance (e.g. zinc), whilst other correlations reversed direction. For example, after accounting for climate and soil properties, higher foliar carbon was associated with lower abundances of all PLFA microbial groups, and most significantly with Gram‐positive bacteria and the two fungal groups (SF and AM), although the relationship with the fungi‐to‐bacteria ratio remained positive (Fig. 5b).

Fewer foliar traits showed consistent associations with the relative abundance of the dominant bacterial phyla, and the direction of these associations varied among phyla, unlike the more uniform patterns observed with broad PLFA microbial groups and ratios (Fig. 5a). Initial correlations showed that several resource‐acquisitive foliar traits (e.g. boron, sulphur and potassium), were associated with the relative abundance of all three dominant bacterial phyla. Specifically, these traits were positively correlated with Acidobacteria and Proteobacteria, but negatively correlated with Actinobacteria. In comparison to the phylum‐level taxa, initial observations indicated that a greater number of foliar traits were correlated with bacterial composition represented by ASVs. For example, both bacterial richness and the first NDMS axes were positively correlated with increases in the concentrations of several resource‐acquisitive foliar traits, including potassium and phosphorus, and negatively correlated with more resource‐conservative traits such as carbon and manganese. The second NDMS axis was associated with fewer foliar traits and was positively correlated with concentrations of resource‐conservative traits such as cellulose and negatively associated with acquisitive traits such as phosphorus (Fig. 5b). As observed for the broad microbial PLFA groups and phylum‐level data, once climate and soil were accounted for, fewer significant correlations remained, and some relationships were reversed. For example, bacterial richness switched from being negatively to positively associated with concentrations of resource‐conservative traits (e.g. lignin and calcium). The first axis of NDMS1 remained characterised by increasing concentrations of the acquisitive trait phosphorus, and the strength and direction of correlations between NDMS axes two and foliar traits remained largely unchanged (Fig. 5b).

### The performance of spectral models for predicting soil microbial community composition

**Fig. 6:**
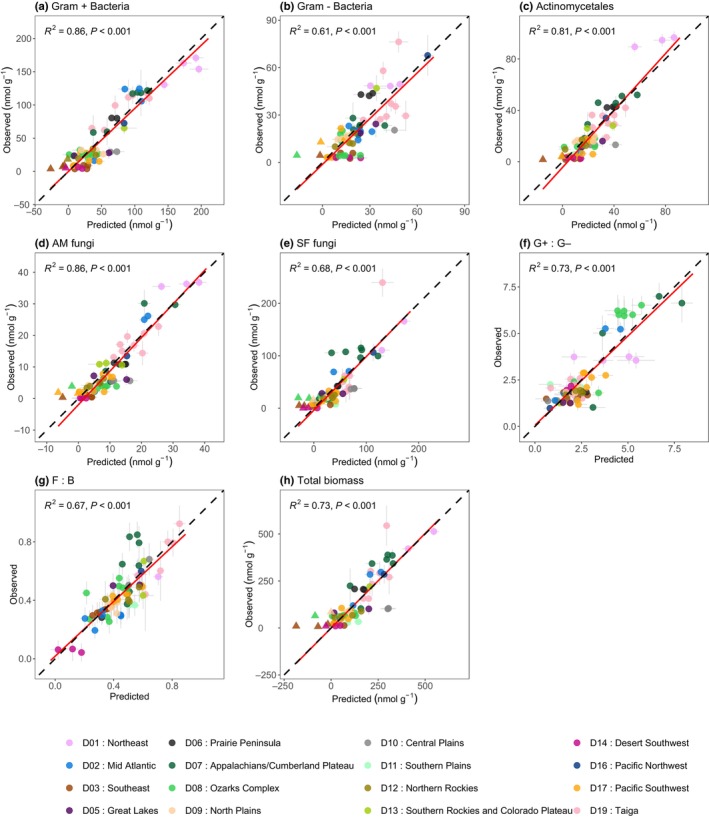
Partial least squares prediction plots for the abundances of broad soil microbial groups as indicated by phospholipid fatty acid biomarkers (a–h). Vertical error bars represent ±1 SD of the observed mean estimate, whereas horizontal error bars represent ±1 SD of the predicted mean estimate (*n* = 200) for each validation plot. The 1 : 1 relationship is shown in black and the line of best fit is shown in red. Triangular data points represent predicted values < 0.

**Fig. 7:**
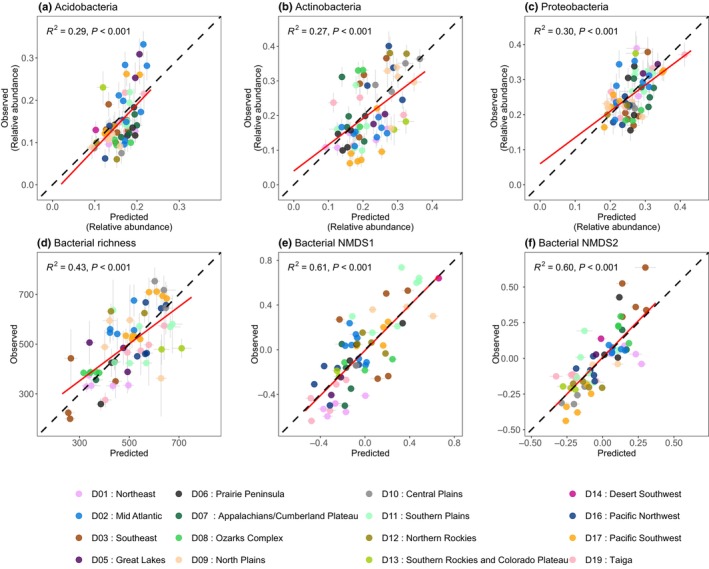
Partial least squares prediction plots for (a–c) the relative abundance of the dominant bacterial phyla, (d) bacterial amplicon sequence variant (ASV) richness and (e, f) the first and second non‐metric multidimensional scaling (NMDS) axes of the bacterial community data. Vertical error bars represent ±1 SD of the observed mean estimate (a–d), whereas horizontal error bars represent ±1 SD of the predicted mean estimate (*n* = 200) for each validation plot. The 1 : 1 relationship is shown in black and the line of best fit is shown in red.

PLSR was used to determine whether canopy spectral reflectance data could be used to predict soil microbial community composition and diversity. Canopy reflectance was able to model the abundance of broad microbial groups and ratios measured via PLFAs with a moderate to good level of predictive accuracy (*R* ^2^ = 0.67–0.86; *P* < 0.001; NRMSE 10.1–14.2%; Fig. 6; Table S9). The abundances of Gram‐positive bacteria, Actinomycetales and AM fungi were modelled with the highest accuracies (*R* ^2^ > 0.8, *P* < 0.001, NRMSE < 11%), although there was some under‐prediction across all models at low PLFA concentrations. The composition of bacterial communities at higher taxonomic resolutions (derived from 16S rRNA sequencing) could also be modelled from canopy reflectance, specifically when characterised by ASVs (Fig. 7d–f). Bacterial community composition gradients (i.e. NMDS axes) were modelled with the highest level of accuracy (*R* ^2^ = 0.6–0.61, *P* < 0.001; NRMSE 12.5–15.5%; Table S9), although richness was also reasonably well modelled (*R* ^2^ = 0.43; *P* < 0.001; NRMSE 15.5%). The relative abundance of bacterial phyla was less well modelled, although all phyla models were still statistically significant (*R* ^2^ = 0.27–0.3; *P* < 0.001; NRMSE > 18%; Fig. 7a–c; Table S9). As a point of contrast, common multispectral indices that are based on several spectral bands as opposed to hundreds of spectral bands, such as the NDVI and NDWI, were only able to explain ≤ 15% of variation in soil microbial community composition and diversity, regardless of how the soil microbiome was characterised (Figs S9, S10).

**Fig. 8:**
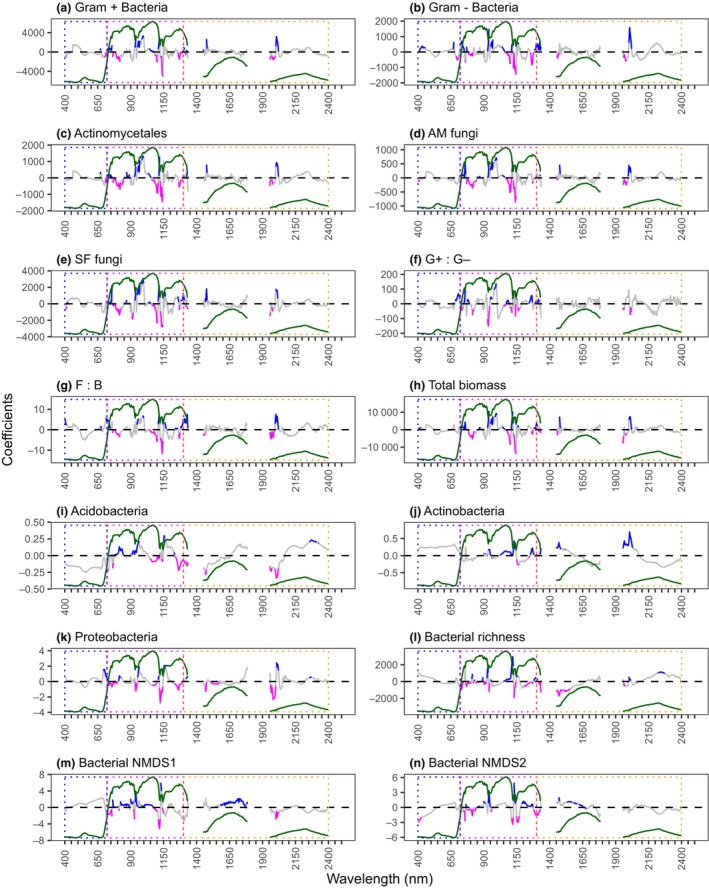
Partial least squares regression (PLSR) model coefficients for (a–h) broad soil microbial groups, (i–k) dominant bacterial phyla, (l) bacterial amplicon sequence variant (ASV) richness and (m, n) the first and second nonmetric multidimensional scaling (NMDS) axes of the bacterial community data. Informative wavelengths (variable importance in projection > 1) are highlighted in blue and magenta (positive and negative coefficients, respectively). Dotted rectangles depict the key regions of the electromagnetic spectrum where blue is visible (VIS); magenta is near infrared (NIR); and orange is the shortwave infrared (SWIR) region. A deciduous forest spectral profile (dark green) is provided in each plot to aid the interpretation of the wavelength regions and spectral absorption features.

Fig. 8 provides information on the wavelengths that have the most significant impact on predicting soil microbial communities, as indicated by the PLSR model coefficients and Variable Importance in Projection (VIP) scores (Fig. S11). For most models, the most important wavelengths were often situated in the near infrared (NIR; 720–1130 nm) and shortwave infrared (SWIR, 1130–2500 nm). Many important wavelengths also coincided with absorption features identifiable in a typical vegetation spectral signature. For example, we observed negative regression coefficients related to known water absorption features in the SWIR at *c*. 1140 nm across all PLFA and ASV models, and Proteobacteria, suggesting that an increase in reflectance in this region of the spectrum (indicative of drier conditions) has a significant effect on soil microbial composition and diversity.

## Discussion

Soil microbial communities are fundamental components of biodiversity and are essential to ecosystem processes such as nutrient cycling, organic matter decomposition and the maintenance of soil health (Delgado‐Baquerizo *et al*., 2018a). Gaining insight into their distribution over large spatial scales is crucial for understanding how microbial community composition and diversity shift across ecosystems and environmental gradients, and how they influence, or are influenced by, broad‐scale changes in vegetation, climate and land use. We provide evidence that imaging spectrometry can capture spatial variation in soil microbial community attributes, including the abundance of key microbial groups, and the diversity and composition of bacterial communities. Our results are in broad agreement with previous studies that point to the potential for imaging spectroscopy to aid in mapping and characterisation of the soil microbiome (e.g. Madritch *et al*., 2020; Sousa *et al*., 2021; Cavender‐Bares *et al*., 2022; Skidmore *et al*., 2022, 2025), but we advance our understanding by demonstrating the wider applicability of the approach across a greater range of diverse ecoregions and land covers at a continental scale. Furthermore, by combining PLFA data with high‐resolution 16S rRNA gene sequencing, together with field‐measured ancillary data, we show that the effectiveness of such models depends not only on how soil microbial communities are characterised (e.g. functional biomass, relative abundance or diversity), but also on how closely plant attributes are linked to soil microbial communities, either directly or indirectly through environmental factors that similarly shape microbial patterns.

We hypothesised that the capacity to predict soil microbial community composition and diversity from canopy reflectance would, at least in part, stem from strong associations between foliar traits linked to plant resource acquisition and conservation strategies; traits that also influence, or are shaped by, underlying soil properties. Indeed, we found that the highest model prediction accuracies aligned with microbial communities whose variation was well explained by a combination of field‐measured foliar traits (Figs 4, 6). Moreover, we found that the importance of foliar traits in explaining soil microbial community composition and diversity was often reinforced by shared responses of both plant traits and microbial communities to underlying environmental gradients such as soil properties and climate (Fig. 4). For example, in comparison to broad taxonomic microbial groups and ratios characterised by PLFA or microbial diversity and composition determined by ASVs, foliar traits (alone or when the variance was shared with other environmental attributes) explained a much smaller proportion of the variation in the relative abundance of dominant bacterial phyla. This was reflected in a weaker performance of the canopy reflectance models for predicting phylum‐level relative abundance. When the influence of climate and soil properties was reduced (via partial correlation) several foliar traits remained significantly correlated with specific microbial community attributes, for example foliar carbon content (e.g. low for G+, SF and AM fungi and high for high fungi‐to‐bacteria ratios), SLA (low for high bacterial richness) and lignin (high for high bacterial richness; Fig. 5). Many of these foliar traits are considered key functional markers, as they relate to soil fertility and the quantity and quality of plant‐derived inputs to the soil (Garnier *et al*., 2004). There was variation in the exact nature of the correlations between specific traits and different microbial community groups, which also suggests that different environmental factors select for different microbial communities and community groups (Averill *et al*., 2021; Malard *et al*., 2022). Nevertheless, strong correlations between foliar traits and soil microbial community attributes characterised by both PLFA and ASVs, albeit often confounded by soil and/or climate gradients, reveal that there are direct correlative links between aboveground plant attributes and soil microbial communities at the continental scale. Both direct and indirect associations can contribute to strong predictive models across large spatial scales, by reflecting the common responses of vegetation and microbial communities to underlying ecological drivers.

We hypothesised that differences in the ability to predict soil microbial community composition and diversity may also be related to the level of taxonomic resolution. Previous work has suggested that soil microbial communities may become more predictable as taxonomic resolution decreases and spatial scale increases, as deterministic processes are likely to shape microbial communities at greater levels of taxonomic and functional aggregation (Averill *et al*., 2021). Our results provide partial support for this hypothesis as crude measures of soil microbial community composition and abundance of microbial groups measured by PLFAs were among the most accurately modelled by canopy reflectance (Fig. 6). This likely reflects the fact that PLFA profiles represent the active microbial biomass and are sensitive to short‐term shifts in plant‐derived inputs such as litter quality (Docherty *et al*., 2015), as observed in the higher correlations between PLFA groups and individual plant traits (Fig. 5). Consequently, our results suggest that PLFA data are useful for capturing broad‐scale functional linkages between aboveground and belowground communities at large spatial scales, particularly where plant traits influence microbial responses through variation in resource quality, quantity, nutrient balance or carbon‐to‐nutrient stoichiometry.

We also expected bacterial communities characterised at the phylum level to be better predicted than those at higher taxonomic resolutions. However, in contrast to this we found that bacterial communities and their diversity characterised by ASVs were more predictable from spectral reflectance at the continental scale than bacterial phyla (Fig. 7). Our results show that although deterministic environmental filtering may have influenced plant traits and bacterial richness and community composition, these processes did not lead to strong covariation between plant attributes and the relative abundance of dominant bacterial phyla (Figs 4, 5). This likely reflects the broad functional and ecological heterogeneity within phyla, which can mask finer‐scale associations with plant communities and is consistent with findings by Delgado‐Baquerizo *et al*. (2018b), who showed that habitat preferences are not strongly linked to phylogeny at coarse levels of taxonomic resolution. For example, dominant bacterial phyla (e.g. Proteobacteria, Actinobacteria and Acidobacteria) often include organisms with diverse ecological roles and environmental preferences. As a result, different members of a single phylum may be related in opposite ways to the same plant trait, which may obscure any consistent plant–microbe covariation at the phylum level and thus their relative abundances are likely shaped by factors beyond the scope of aboveground spectral cues. By contrast, bacterial richness and ordination‐based composition metrics capture shifts at finer taxonomic resolution and therefore may be more sensitive to deterministic environment–plant–microbe linkages. Dominant phyla may maintain high overall abundance across environments, while subtle changes within them account for most of the ecological signal. Consequently, even under deterministic processes, plant traits may not relate to phylum‐level abundances but they may be indicative of change in community structure.

We used the full canopy reflectance spectrum to model soil microbial communities because plant functions and strategies directly influence or are closely linked to the optical properties of plant canopies (Kattenborn *et al*., 2019; Kattenborn & Schmidtlein, 2019). The wavelengths that accounted for most of the variation in our spectral models were primarily found in the NIR and SWIR regions, which are known to reflect key foliar structural, physiological and chemical traits (Kokaly *et al*., 2009; Ollinger, 2011; Fig. 8). Interpreting the physical meaning of specific wavelengths identified by our models is challenging as many foliar traits relevant to soil microbial communities, including those measured in our field data, lack distinct spectral features and often do not show linear relationships with reflectance (Kokaly *et al*., 2009; Ollinger, 2011). For instance, lignin and cellulose concentrations have non‐linear effects on spectral reflectance. Both compounds exhibit strong absorption features in the SWIR region (1500–2500 nm), but increasing concentrations also affect leaf structural properties, altering light scattering (especially in the NIR) and thus influencing reflectance across multiple spectral regions (Wang *et al*., 2020). Wavelengths near known water absorption features in the SWIR (e.g. *c*. 1150 nm) were important in many soil microbial community models, suggesting that canopy moisture variations may help predict soil microbial community composition and diversity. Previous research across the western US identified SM as a key driver of canopy water content dynamics (Lyons *et al*., 2021). Since both our results and those of others indicate that SM strongly influences soil microbial communities (Brockett *et al*., 2012; Guenet *et al*., 2012; Waldrop *et al*., 2017), it is likely that the observed changes in canopy reflectance are also associated with variations in SM. The importance of broad spectral regions (i.e. NIR and SWIR) in many of our predictive models may suggest that more widely available multispectral imagery, which contains fewer spectral bands, may be equally as useful for predicting key soil microbial community attributes. However, linear models based on standard multispectral indices such as the NDVI (a general proxy for biomass/productivity) and the NDWI (a general indicator of canopy moisture) performed poorly on the same dataset (Figs S9, S10). These results suggest that hyperspectral data provide more informative signals for predicting soil microbial community attributes and highlight the benefits of hyperspectral data in ecological research (Ustin *et al*., 2004; Kokaly *et al*., 2009; Serbin & Townsend, 2020; Cavender‐Bares *et al*., 2025).

At the canopy scale, reflectance is also affected by canopy architecture, understory vegetation, and in some cases, the underlying substrate (Ollinger, 2011). While our results show that negative PLSR coefficients in the NIR corresponded with known water absorption features, several models also identified important NIR wavelengths with positive coefficients associated with increased reflectance (Fig. 8). High NIR reflectance results from multiple scattering within mesophyll tissue at the leaf level as well as canopy structural differences such as leaf and branch density, total green vegetation cover and vegetation dry matter content (Tucker, 1979). Although we vector‐normalised our imagery and applied an NDVI threshold (i.e. < 0.4) to remove sparsely vegetated and shadowed canopies (Figs S1, S2), it is possible that our PLSR regression models may be capitalising on both direct effects of pigments and water on plant reflectance (manifested as specific absorption features) and indirect effects arising from associations between plant physiological and chemical traits, and structural features that influence canopy reflectance (Townsend *et al*., 2013; Lepine *et al*., 2016; Wang *et al*., 2022). Previous research has demonstrated the potential of LiDAR‐derived measures of canopy structural diversity to infer variations in soil microbial composition (Lang *et al*., 2023). However, such relationships have so far only been observed in hardwood forests, as current aerial LiDAR technology cannot effectively measure the structure of low‐stature herbaceous vegetation (Li *et al*., 2021).

An additional challenge of linking remotely sensed data with soil microbial community attributes is the vast differences in scale between small spatial scale observations, usually at the level of individual soil cores, and large spatial scale observations from airborne or satellite platforms. Whilst heterogeneity belowground within the soil microbiome is several orders of magnitude greater than in aboveground plant canopies (Bardgett & van der Putten, 2014), we were still able to predict soil microbial community attributes using *in situ* and airborne data aggregated to 20 m pixels (i.e. to match sampling within the NEON plots); similarly to Skidmore *et al*. (2025) who used 30 m spaceborne imaging spectroscopy data to map soil microbial diversity across temperate European forests and Wang *et al*. (2022) who used plot‐level aggregated spectral reflectance to map foliar traits across several NEON sites. Aggregating soil microbial communities across larger spatial scales has also been shown to enhance the predictability of certain bacterial and fungal groups, likely due to the influence of deterministic environmental filtering (Averill *et al*., 2022). Furthermore, at the continental scale, links between aboveground and belowground communities have a greater chance of being observed given that differences in plant traits and attributes are likely to be significant (Delgado‐Baquerizo *et al*., 2018a). Predicting soil microbial communities from canopy reflectance data alone may be more challenging as the spatial extent across which predictions are made decreases. Further work is thus required to look at how predictability varies across spatial and temporal extents, both within and between ecosystems.

We used PLSR models to predict microbial community composition and diversity from canopy spectral reflectance. Whilst PLSR models are often used in the prediction of ecological attributes from remotely sensed data, empirical models are often criticised for their lack of transferability. Insufficient data prevented us from exploring the transferability of our soil microbial attribute models using approaches such as cross‐domain validation (Wang *et al*., 2020; Zhang *et al*., 2022) or testing our models on truly independent datasets (i.e. from plots not within the NEON network), and to explicitly link field‐measured traits with reflectance and soil microbial communities at scale. Recent studies also indicate that foliar trait‐based PLSR models work best when training data are representative of the data in the validation sample (Nakaji *et al*., 2019; Wang *et al*., 2020; Ji *et al*., 2024), and it is likely that the same holds true for our PLSR models. Consequently, there is a clear necessity to continue enhancing efforts to develop extensive training datasets for imaging sensors, to ensure reliable model performance over large spatial extents.

### Conclusions

Although remote sensing cannot replace field‐based measurements, our results show that canopy reflectance can reliably capture variation in the composition and diversity of soil microbial communities at continental scales. This predictive capacity is underpinned by plant attributes, which influence microbial communities directly and also reflect shared responses to underlying edaphic and climatic gradients. We further show that model accuracy depends on taxonomic resolution, peaking when microbial communities are characterised at levels that correspond to coherent habitat preferences detectable through canopy spectral signatures. Models based on full‐spectrum hyperspectral data consistently outperform those using simple vegetation indices such as NDVI and NDWI, highlighting the current and future importance of imaging spectroscopy in ecological research. The upcoming deployment of multiple satellite imaging spectrometers, including ESA's Copernicus Hyperspectral Imaging Mission for the Environment (CHIME) and NASA's Surface Biology and Geology, both planned to launch in 2028, combined with expanded field sampling, offers an unprecedented opportunity to map soil microbial biogeography across diverse landscapes and accelerate the development of rapid, accurate and cost‐effective ecosystem monitoring frameworks.

## Competing interests

None declared.

## Author contributions

AH and RDB conceived the study. AH processed the data, carried out the analysis and led the writing of the original draft. RDB contributed to the writing and revision of the manuscript.

## Disclaimer

The New Phytologist Foundation remains neutral with regard to jurisdictional claims in maps and in any institutional affiliations.

## Supporting information


**Fig. S1** Mean vector normalised canopy reflectance derived from the images across domains.
**Fig. S2** Mean vector normalised canopy reflectance derived from the images across land cover classes.
**Fig. S3** Correlations among foliar traits, soil properties and climate attributes.
**Fig. S4** Distribution of foliar traits grouped by NEON domain.
**Fig. S5** Distribution of soil properties grouped by NEON domain.
**Fig. S6** Distribution of PLFA soil microbial community groups grouped by NEON domain.
**Fig. S7** Distribution of bacterial phyla and diversity grouped by NEON domain.
**Fig. S8** Ordination plots (PCA and NMDS) across NEON land cover classes.
**Fig. S9** Simple linear regression plots based on the Normalised Difference Vegetation Index (NDVI).
**Fig. S10** Simple linear regression plots based on the Normalised Difference Water Index (NDWI).
**Fig. S11** Partial least squares regression Variable Importance in Projection (VIP) plots.
**Methods S1** NEON soil sampling.
**Methods S2** Aggregating data to the plot scale.
**Methods S3** NEON foliar trait sampling.
**Table S1** Description of NEON datasets.
**Table S2** Description of sampled NEON sites.
**Table S3** Description of measured NEON variables.
**Tables S4–S7** The relative contributions of variables to PCA axes.
**Table S8** Significance results from variation partition modelling.
**Table S9** Summary statistics for partial least squares regression modelling.
Please note: Wiley is not responsible for the content or functionality of any Supporting Information supplied by the authors. Any queries (other than missing material) should be directed to the *New Phytologist* Central Office.

## Data Availability

The specific NEON datasets used can be accessed using the following links: DP1.10104.001 Soil microbe biomass: https://doi.org/10.48443/rwbj-ry66; DP1.10081.001 Soil microbe community composition: https://doi.org/10.48443/p0ge-z118; DP1.10086.001 Soil physical and chemical properties, periodic: https://doi.org/10.48443/0phb-j505; DP3.30006.001 Spectrometer orthorectified surfacedirectional reflectance: https://doi.org/10.48443/49kq-8q12; DP1.10026.001 Plant foliar traits: https://doi.org/10.48443/tmrs-fb32.
