# Supplementary material for: Canopy reflectance as a predictor of soil microbial community composition and diversity at a continental scale
Source: New Phytol. 2025 Nov 19;249(2):829–47. doi: 10.1111/nph.70720 (PMC12712435; doi:10.1111/nph.70720)
Supplement: Supplementary file 1 — Fig. S1 Mean vector normalised canopy reflectance derived from the images across domains. Fig. S2 Mean vector normalised canopy reflectance derived from the images across land cover classes. Fig. S3 Correlations among foliar traits, soil properties and climate attributes. Fig. S4 Distribution of foliar traits grouped by NEON domain. Fig. S5 Distribution of soil properties grouped by NEON domain. Fig. S6 Distribution of PLFA soil microbial community groups grouped by NEON domain. Fig. S7 Distribution of bacterial phyla and diversity grouped by NEON domain. Fig. S8 Ordination plots (PCA and NMDS) across NEON land cover classes. Fig. S9 Simple linear regression plots based on the Normalised Difference Vegetation Index (NDVI). Fig. S10 Simple linear regression plots based on the Normalised Difference Water Index (NDWI). Fig. S11 Partial least squares regression Variable Importance in Projection (VIP) plots. Methods S1 NEON soil sampling. Methods S2 Aggregating data to the plot scale. Methods S3 NEON foliar trait sampling. Table S1 Description of NEON datasets. Table S2 Description of sampled NEON sites. Table S3 Description of measured NEON variables. Tables S4–S7 The relative contributions of variables to PCA axes. Table S8 Significance results from variation partition modelling. Table S9 Summary statistics for partial least squares regression modelling. Please note: Wiley is not responsible for the content or functionality of any Supporting Information supplied by the authors. Any queries (other than missing material) should be directed to the New Phytologist Central Office. [file NPH-249-829-s001.pdf]

New Phytologist

New Phytologist **Supporting Information**

Article title: Canopy reflectance as a predictor of soil microbial community composition and diversity at a continental scale
Authors: Angela Harris and Richard D. Bardgett
Article acceptance date: 14 October 2025

The following Supporting Information is available for this article:

Methods S1 Soil sampling is conducted annually at a minimum of one site per domain with frequencies ranging from once per year in regions with short growing seasons to up to three times per year in regions with longer growing seasons. Sampling occurs during the historical peak of vegetation greenness at each site. Samples were collected using soil cores (~ 7 cm diameter) from the upper 30 cm of the soil surface, with organic and mineral horizons sampled independently to preserve distinct soil microbial communities. Soil samples collected for C and N concentrations undergo initial processing at the domain laboratory, consisting of sieving, air-drying and sub-sampling. Once prepared, the samples are sent to contracted laboratory facilities for analysis. Additional subsamples are analysed for pH and moisture at the domain laboratory, and a separate portion is preserved for archival purposes. For microbial analysis subsamples are either put on dry ice in the field (for microbial genetic analysis e.g. 16S), or kept field moist and then prepped in the lab (for microbial biomass analysis via PLFA). Samples are shipped to contracted facilities within 24 hrs for processing and analysis. Additional details on NEON's soil biogeochemical and microbial sampling protocol and procedures can be found in Stanish (2024).

Methods S2 To facilitate integration of field data with airborne imagery, we employed a spatial scaling approach using Bayesian models to estimate plot-level Bayesian average values for foliar traits, soil properties, soil microbial biomass, soil community composition at the phylum-level and bacterial richness (Averill *et al.*, 2021). To estimate mean relative abundances of bacterial phyla we used a Dirichlet distribution model to estimate the mean abundance of multiple phylum simultaneously, whilst accounting for covariance among phylum abundances, since compositional data within a sample sum to 1 (Pawlowsky-Glahn *et al.*, 2015). All Bayesian models were fit using 3 Markov chains with 2,000 adaptive iterations, 5,000 burn-in and 30,000 sample iterations drawn from their respective distributions. We removed plots where the Markov chains failed to converge (Gelman-Rubin diagnostic <1.1 and effective sample size > 400). Aggregation was implemented using the runjags package for R statistical software (Denwood, 2016), to fit JAGS models (Plummer, 2003).

Methods S3 The foliar sampling protocol involved the collection of samples from 3 plant individuals located within the 20 m x 20 m core of each 40 x 40 m plot. For a tree plot, three trees were sampled; for a grass plot three clip samples were collected. In the absence of species-level trait measurements per plot, we aggregated all trait measurements within each plot to obtain a

mean trait value per plot. We did not use foliar data collected post 2019 as the protocol changed from measuring individuals per plot to representative individuals per site. Additional details regarding the NEON's protocol and procedures for canopy foliage sampling can be found in Weintraub-Leff (2025).

Fig. S1 Spectral reflectance profiles obtained from the Airborne Observation Platform (AOP) across each domain. The ribbon in each plot represents the range of vector normalised spectra within that domain

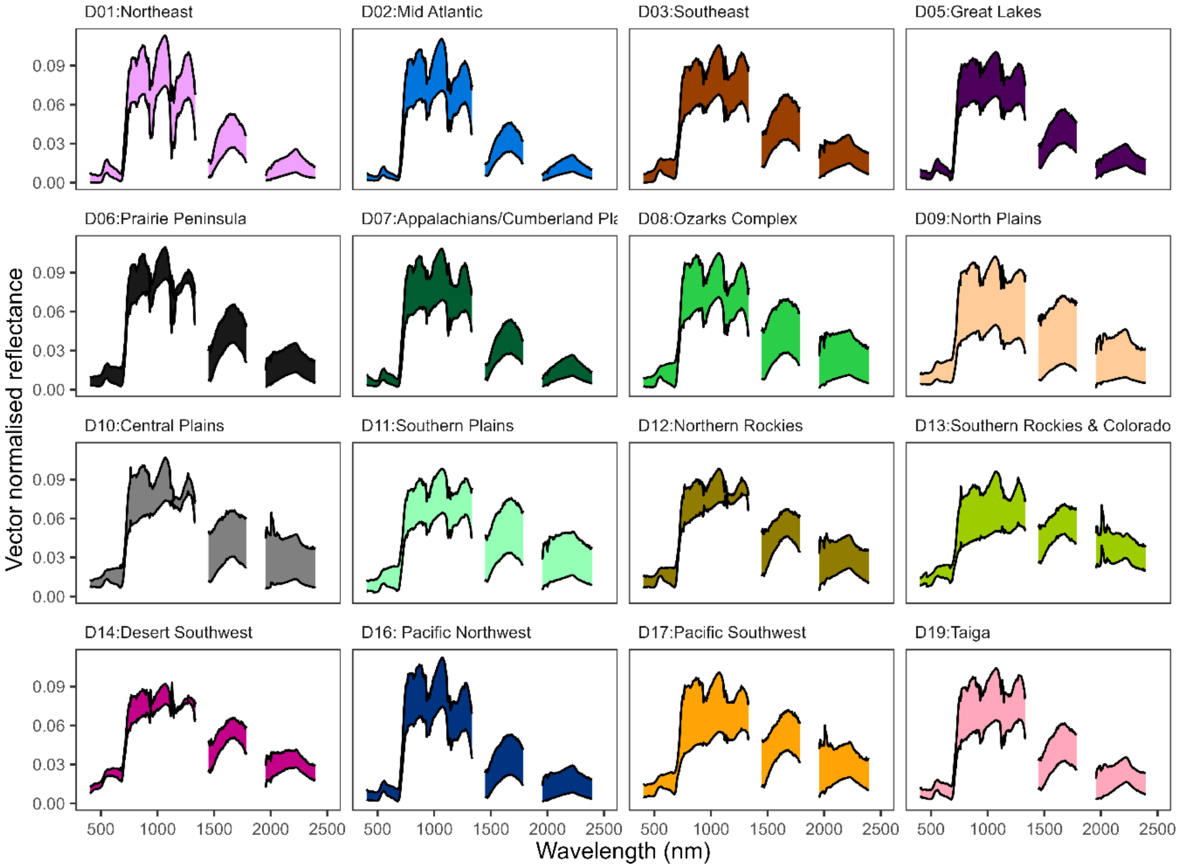

Fig. S2 Spectral reflectance profiles obtained from the Airborne Observation Platform (AOP) across NEON defined land cover class. The ribbon in each plot represents the range of vector normalised spectra within that land cover class.

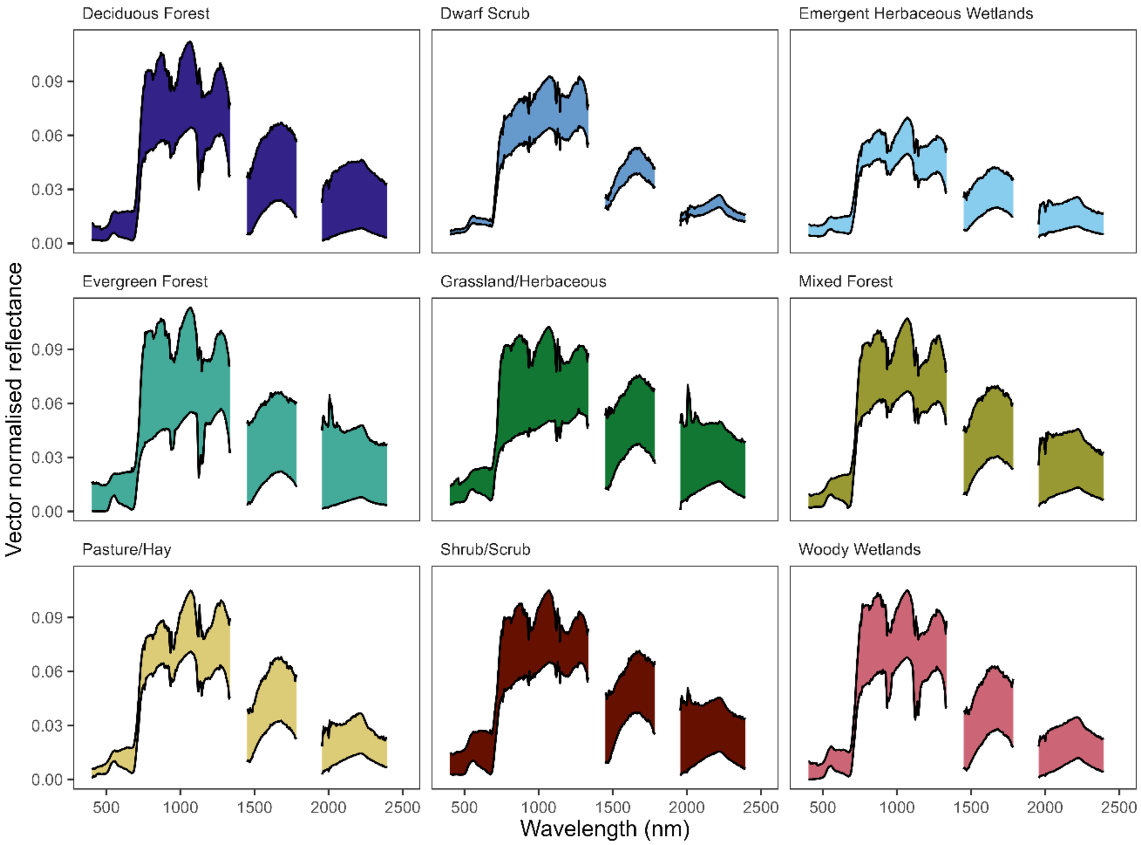

Fig. S3 Correlations among (a) foliar traits (n = 104), (b) soil and climate attributes (n = 182), and (c) all field measured variables (n = 96) using spearman rank correlation with complete cases. Only correlations $p < 0.05$ are shown.

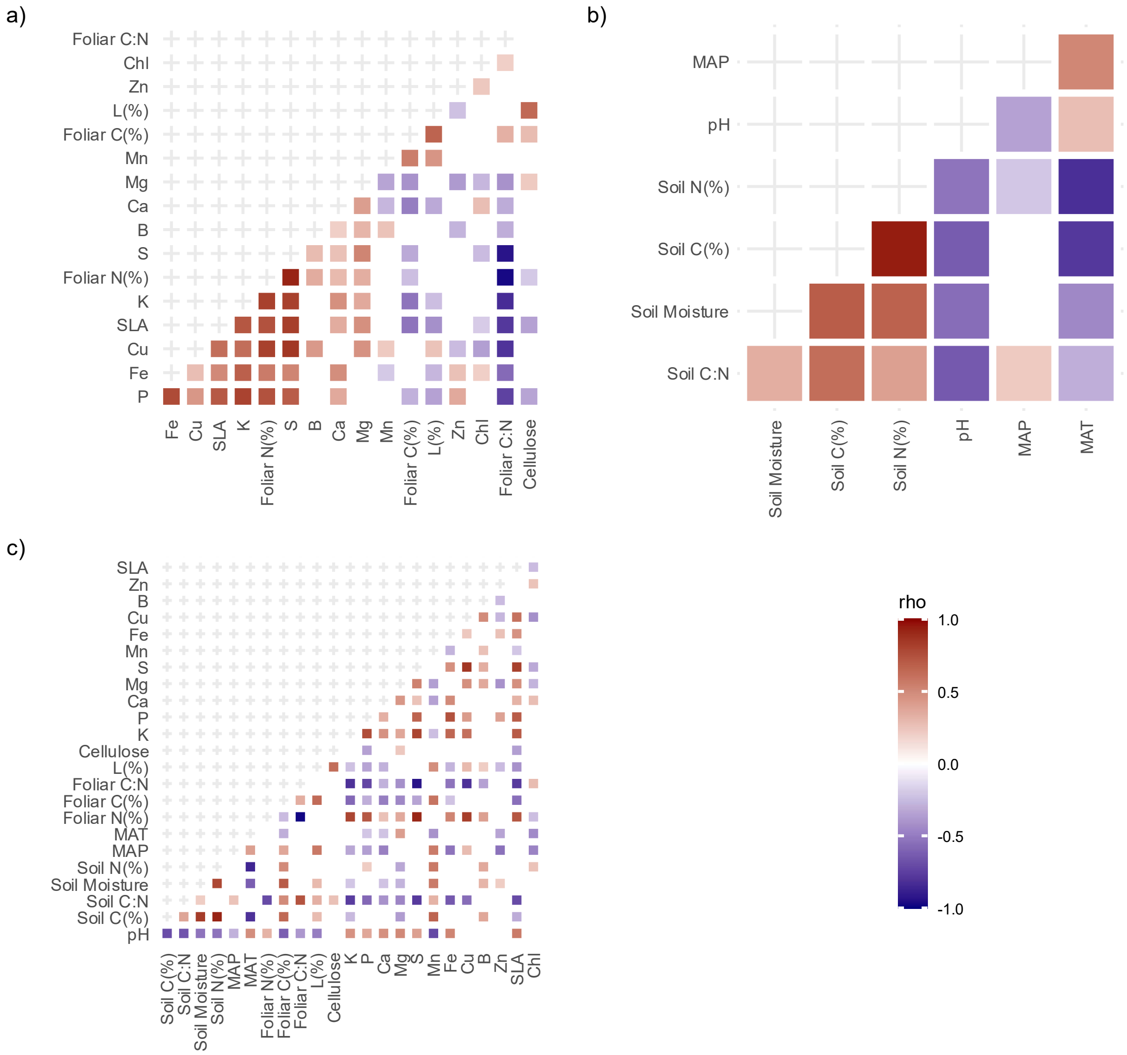

New Phytologist

Fig. S4 Distribution of foliar traits measured across sampled plots and grouped by NEON domain. The black dots represent values for individual plots sampled within a given domain, horizontal lines represent the median, whiskers indicate the variability outside the interquartile range.

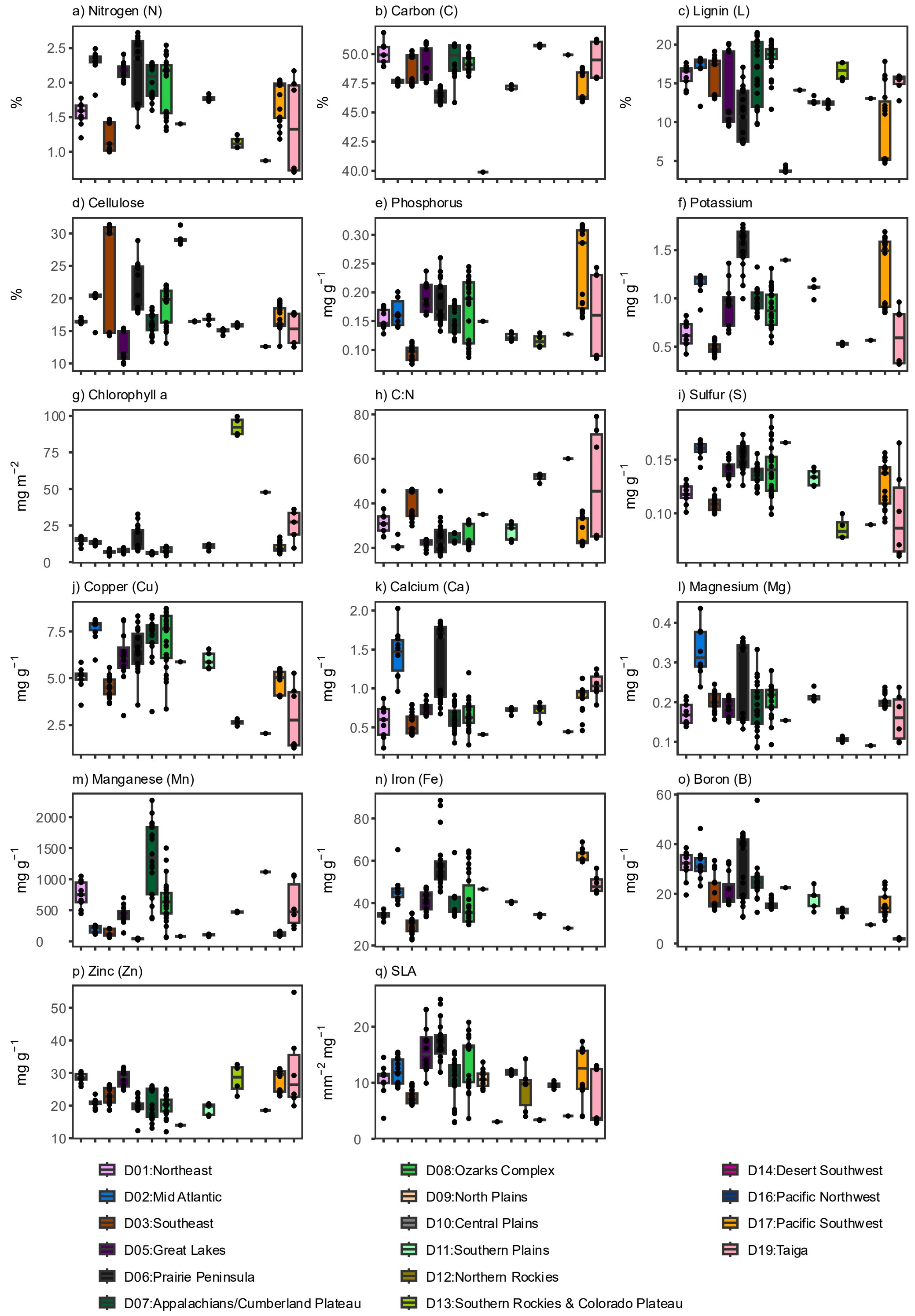

Fig. S5 Distribution of soil properties and climate variables measured across sampled plots and grouped by NEON domain. The black dots represent values for individual plots sampled within a given domain, horizontal lines represent the median, whiskers indicate the variability outside the interquartile range.

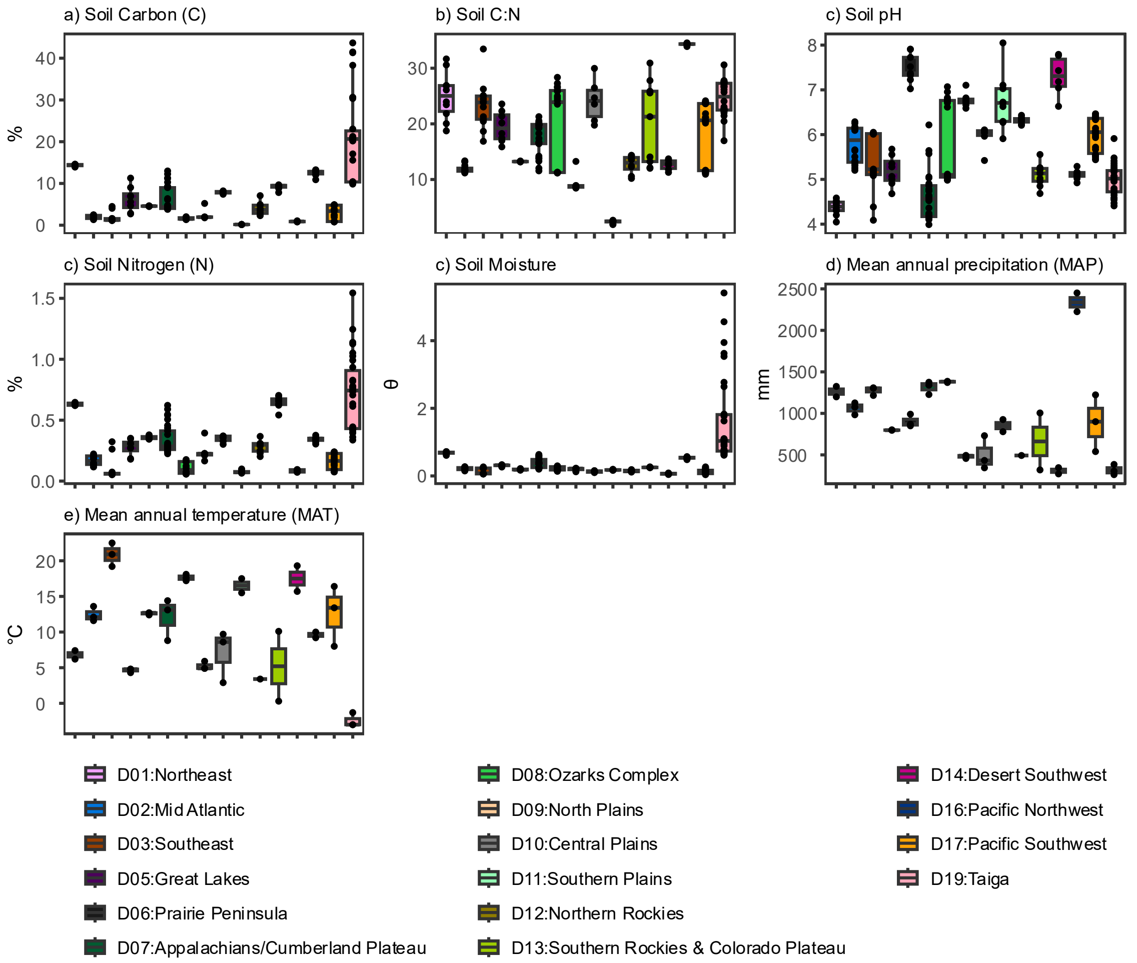

Fig. S6 Distribution of broad soil microbial groups measured by summing concentrations of phospholipid fatty acids (PLFA) across sampled plots and grouped by NEON domain. The black dots represent values for individual plots sampled within a given domain, horizontal lines represent the median, whiskers indicate the variability outside the interquartile range.

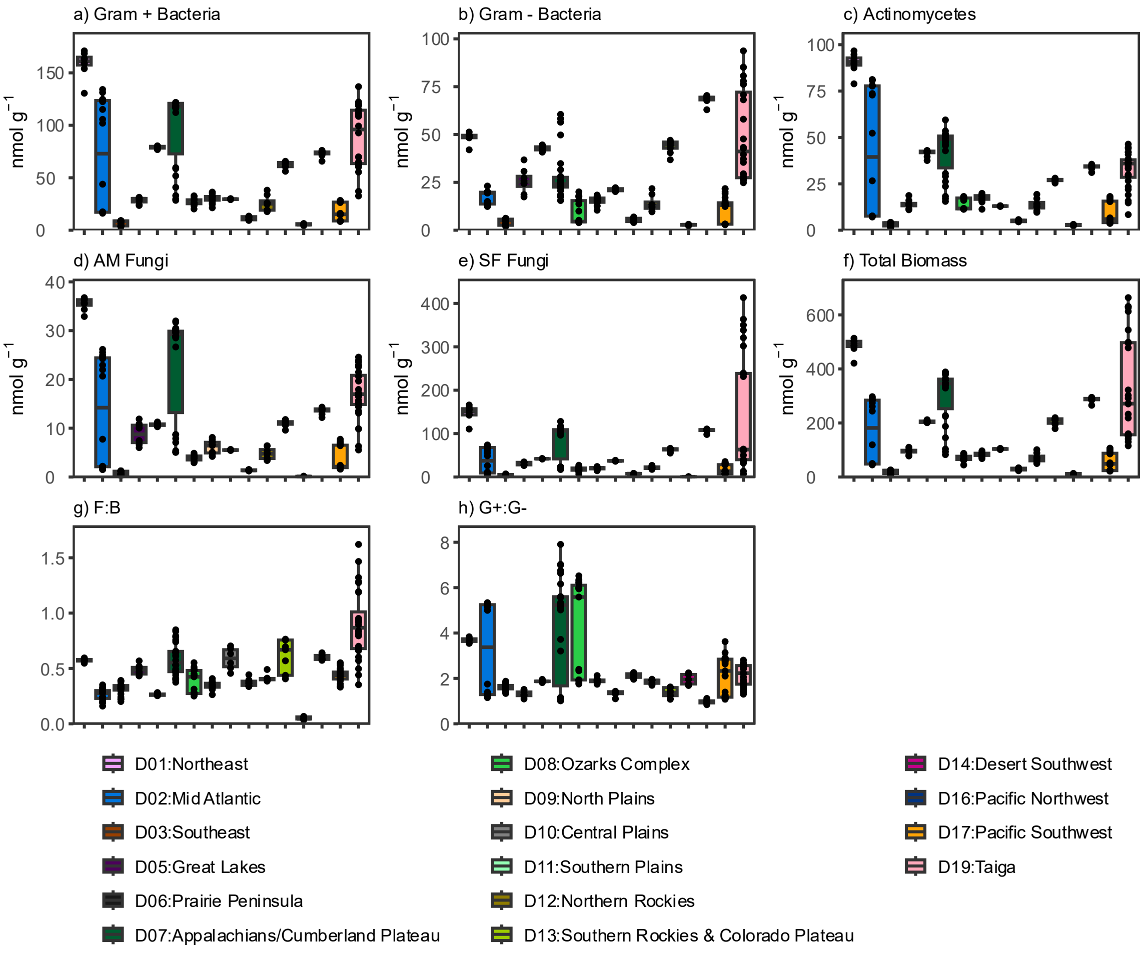

New Phytologist

Fig. S7 Distributions of (a-d) the relative abundance of the most abundant bacterial phyla and (e) bacterial amplicon sequence variant (ASV) richness, measured across sampled plots and grouped by NEON ecoclimatic domain. The black dots represent values for individual plots sampled within a given domain, horizontal lines represent the median, whiskers indicate the variability outside the interquartile range.

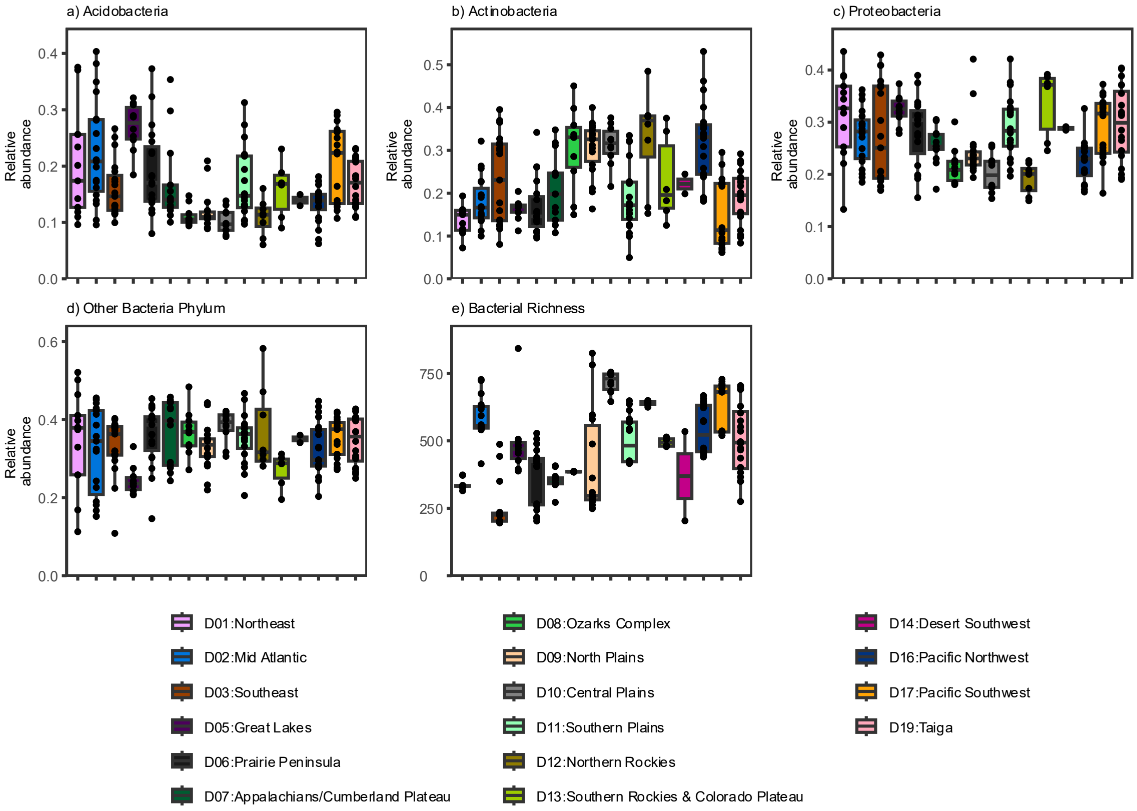

Fig. S8 Ordinations (PCA and NMDS) of a) PLFA groups (n = 232), b) dominant bacterial phyla (n = 225), c) foliar traits (n = 146), d) soil properties (n = 217) and e) bacterial community composition (ASVs n = 255) and ASV richness shown as contour lines. Points are coloured according to NEON land cover class.

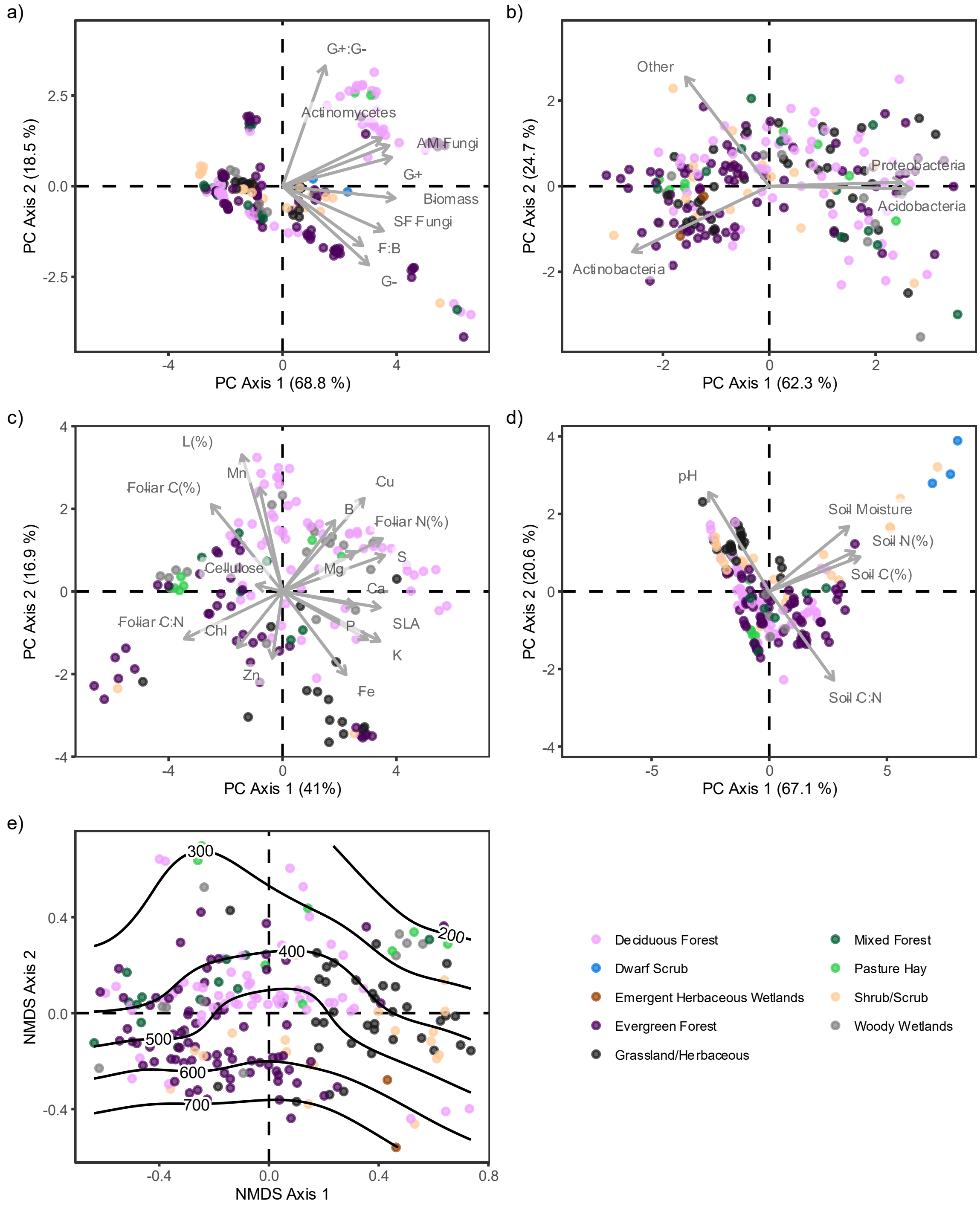

Fig. S9 Normalised Difference Vegetation Index (NDVI) regression prediction plots for (a-h) abundances of broad soil microbial groups; (i-k) relative abundances of the dominant bacterial phyla; (l) bacterial amplicon sequence variant (ASV) richness and (m) the first NMDS axis of the bacterial community data and n) the second NMDS axis of the bacterial community data. Vertical error bars represent +/- 1 standard deviation of the observed mean estimate (a-l), whereas horizontal error bars represent +/- 1 standard deviation of the predicted mean estimate (n=200) for each validation plot. The 1:1 relationship is show in black and the line of best fit is shown in red. Triangular data points represent predicted values < 0.

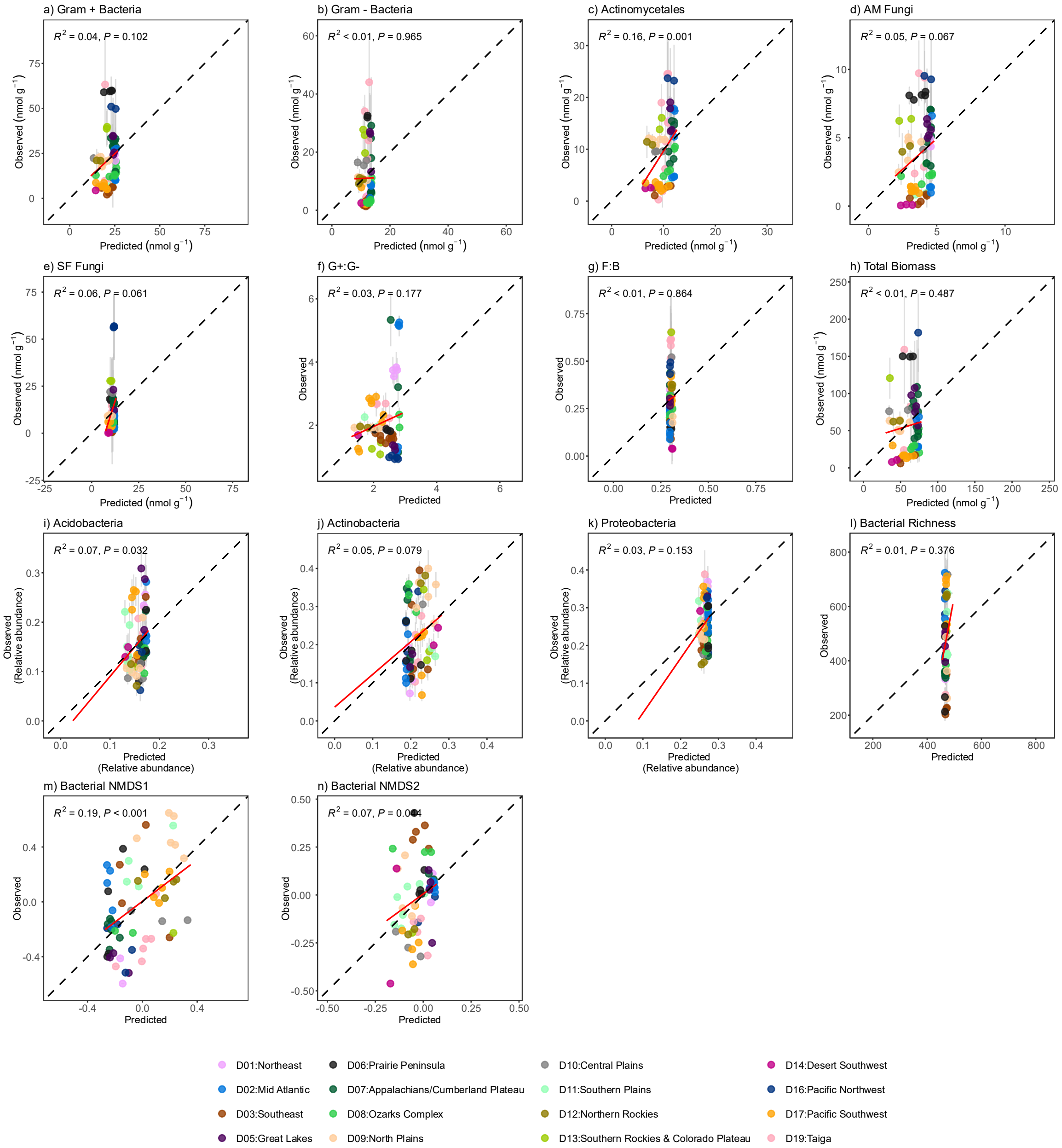

Fig. S10 Normalised Difference Water Index (NDWI) regression prediction plots for (a-h) abundances of broad soil microbial groups; (i-k) relative abundances of the dominant bacterial phyla; (l) bacterial amplicon sequence variant (ASV) richness, (m) the first NMDS axis of the bacterial community data and n) the second NMDS axis of the bacterial community data. Vertical error bars represent +/- 1 standard deviation of the observed mean estimate (a-l), whereas horizontal error bars represent +/- 1 standard deviation of the predicted mean estimate (n=200) for each validation plot. The 1:1 relationship is show in black and the line of best fit is shown in red. Triangular data points represent predicted values < 0.

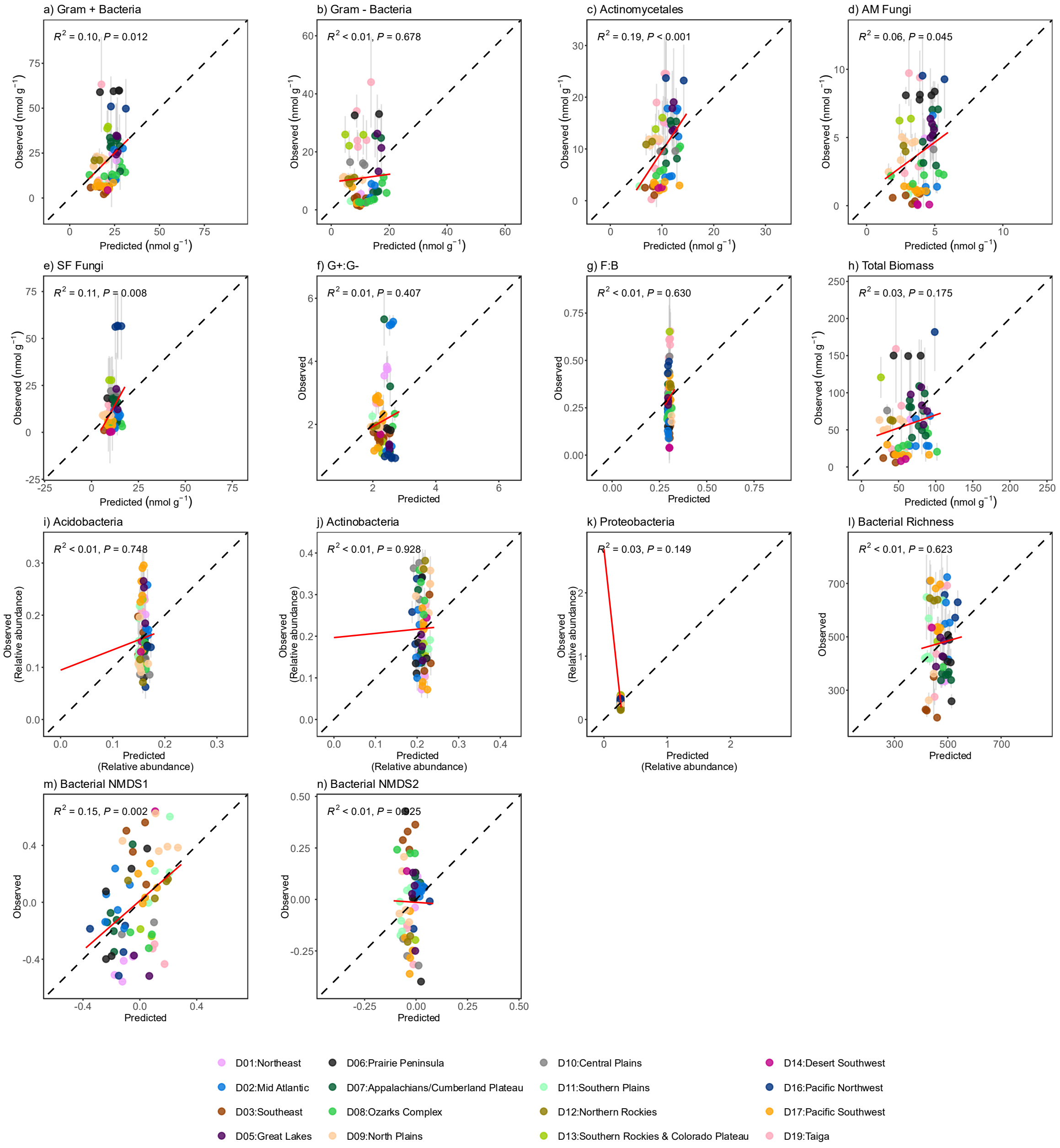

Fig. S11 . Mean partial least squares regression (PLSR) model variable importance in projection (VIP) scores (n=200), for selected PLSR models of the soil microbial community (with +/- 1 standard deviation bands in blue). VIP scores below the horizontal line (<1) indicate parts of the spectrum that are not informative for microbial community predictions. Dotted rectangles depict the key regions of the electromagnetic spectrum where blue is visible (VIS); magenta is near infrared (NIR); and orange is the shortwave infrared region (SWIR).

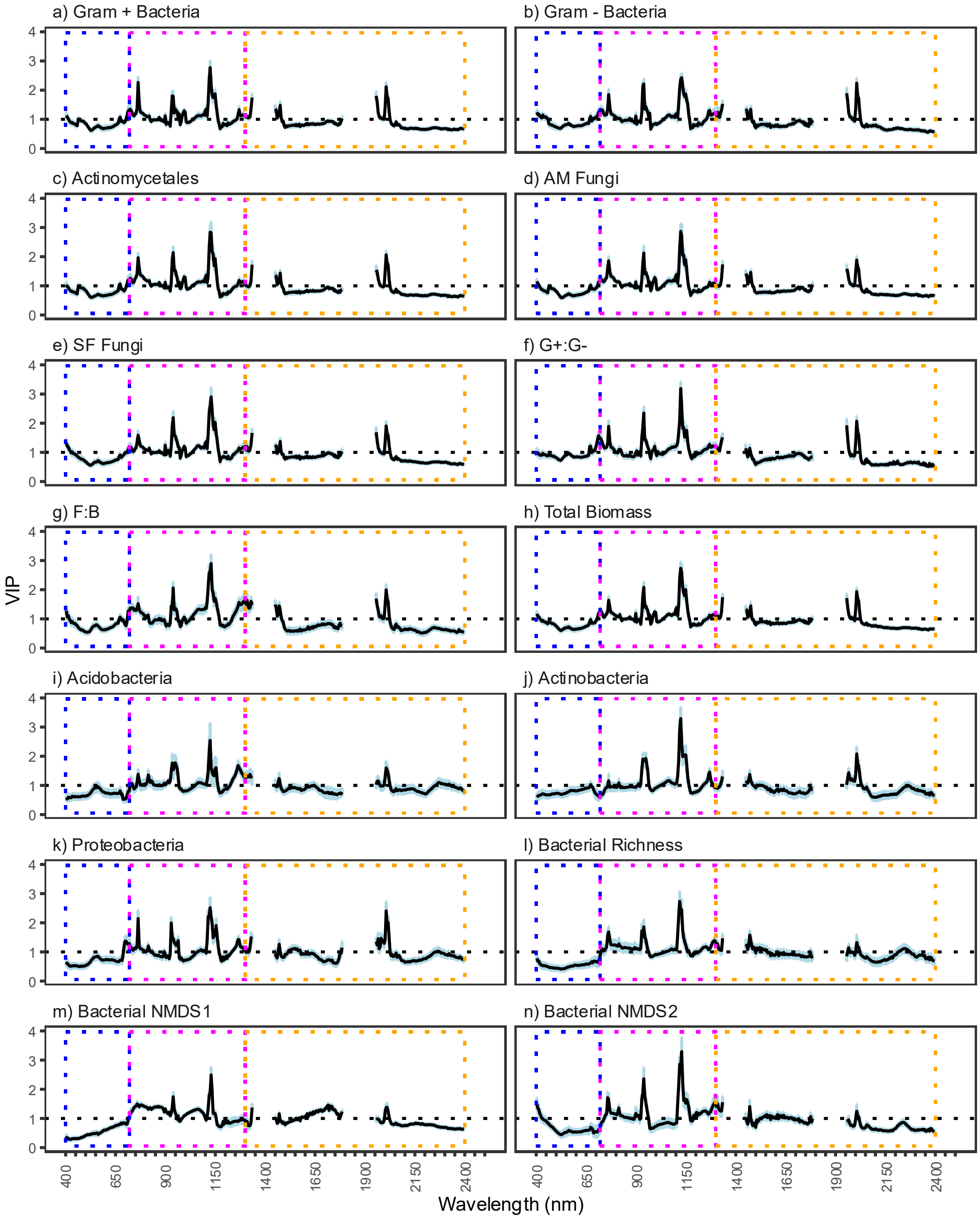

Table S1 A description of the different NEON datasets used in this study.

Dataset ID	Dataset Name	DOI	Description	Sampling Frequency	Data availability*
DP1.10104.001	Soil microbe biomass (PLFA)	https://doi.org/10.48443/rwbj-ry66	Quantitative abundance of microbes in soil samples	Every yr. core sites; Every 5 yrs. for other sites	2017-2021
DP1.10081.001	Soil microbe community composition (16s rRNA gene sequencing)	https://doi.org/10.48443/p0ge-z118	Counts and relative abundances of archaeal, bacteria and fungal taxa observed in soil microbial communities	Every yr. core sites; Every 5 yrs. for other sites	2014-2018
DP1.10086.001	Soil physical and chemical properties, periodic	https://doi.org/10.48443/0phb-j505	Soil physical and chemical properties from the top 30 cm of the profile from periodic soil core collections.	Every 5 yrs for total organic C and total N and every soil sampling event for pH and moisture	2013-2021
DP3.30006.001	Spectrometer orthorectified surface directional reflectance - mosaic	https://doi.org/10.48443/49kq-8q12	Orthorectified surface reflectance computed from the NEON Imaging Spectrometer per pixel. Flight lines over a given flight are mosaicked into 1km x 1 km tiles with a spatial resolution of 1 m	Every yr., weather dependent	2013-2021
DP1.10026.001	Plant foliar traits	https://doi.org/10.48443/tmrs-fb32	Traits of sun-lit canopy plants reported at the level of the individual (woody plants) or community (herbaceous plants)	Every 5 yrs.	2013-2021

*Data availability refers to the availability of data at the time the data were accessed

Table S2 Description of sampled NEON sites.

Domain	Domain Name	Site Code	Site Name
D01	Northeast	BART	Bartlett Experimental Forest
		HARV	Harvard Forest & Quabbin Watershed
DO2	Mid Atlantic	SCBI	Smithsonian Conservation Biology Institute
		SERC	Smithsonian Environmental Research Centre
		BLAN	Blandy Experimental Farm
D03	Southeast	JERC	The Jones Centre at Ichauway
		DSNY	Disney Wilderness Preserve
		OSBS	Ordway-Swisher Biological Station
D05	Great Lakes	UNDE	University of Notre Dame Environmental Research Centre
		STEI	Steigerwaldt-Chequamegon
D06	Prairie Peninsula	KONZ	Konza Prairie Biological Station
		UKFS	University of Kansas Field Station
D07	Appalachians & Cumberland Plateau	ORNL	Oak Ridge
		MLBS	Mountain Lake Biological Station
		GRSM	Great Smoky Mountains National Park
DO8	Ozarks Complex	TALL	Talladega National Forest
		DELA	Dead Lake
		LENO	Lenoir Landing
DO9	Northern Plains	NOGP	Northern Great Plains Research Laboratory
		WOOD	Chase Lake National Wildlife Refuge
D10	Central Plains	RMNP	Rocky Mountains
D11	Southern Plains	CLBJ	Lyndon B. Johnson National Grassland
		OAES	Marvin Klemme Range Research Station
D12	Northern Rockies	YELL	Yellowstone National Park
D13	Southern Rockies & Colorado	NIWO	Niwot Ridge
D14	Desert Southwest	SRER	Santa Rita Experimental Range
D16	Pacific Northwest	ABBY	Abby Road
		WREF	Wind River Experimental Forest
D17	Pacific Southwest	TEAK	Lower Teakettle
		SOAP	Soaproot Saddle
		SJER	San Joaquin Experimental Range
D19	Taiga	BONA	Caribou-Poker Creeks Research Watershed
		DEJU	Delta Junction
		HEAL	Healy

Table S3 List of NEON measured variables and their allocated groups for variation partition modelling.

Group of variables	Property/Attribute	Unit	Details
**Foliar**	N	%	Percent nitrogen in a sample on a dry weight basis.
	C	%	Percent carbon in a sample on a dry weight basis.
	C:N	-	Ratio of carbon to nitrogen concentration in a sample on a dry weight basis
	L	%	Percent lignin on a dry mass basis.
	Cellulose	%	Percent cellulose on a dry mass basis
	K	%	Concentration of potassium in plant foliage sample on a dry mass basis
	P	%	Concentration of phosphorus in plant foliage sample on a dry mass basis
	Ca	%	Concentration of calcium in plant foliage sample on a dry mass basis
	Mg	%	Concentration of magnesium in plant foliage sample on a dry mass basis
	S	%	Concentration of sulphur in plant foliage sample on a dry mass basis
	Mn	mg kg^{-1}	Concentration of manganese in plant foliage sample on a dry mass basis
	Fe	mg kg^{-1}	Concentration of iron in plant foliage sample on a dry mass basis
	Cu	mg kg^{-1}	Concentration of copper in plant foliage sample on a dry mass basis
	B	mg kg^{-1}	Concentration of boron in plant foliage sample on a dry mass basis
	Zn	mg kg^{-1}	Concentration of zinc in plant foliage sample on a dry mass basis

	SLA	$m^2 g^{-1}$	Specific leaf area obtained by dividing leaf area by dry mass
	Chl	$mg\ m^{-2}$	Concentration of chlorophyll *a* in plant foliage
**Soil**	pH		pH value of soil measured in water solution
	C	%	Percent organic carbon in a sample on a dry weight basis
	N	%	Percent nitrogen in a sample on a dry weight basis
	C:N	-	Ratio of carbon to nitrogen concentration in a sample on a dry weight basis
	Moisture	-	Gravimetric water content of soil in grams of water per gram dry soil
**Climate**	MAP	mm/year	Mean annual precipitation
	MAT	°C/year	Mean annual temperature
**Land cover**	Land cover	-	National land cover database (NLCD) classification across the U.S used to categorise land cover types based on satellite imagery.

Table S4 Variable contributions to PLFA-based soil microbial community principal components (PC1, PC2 and PC3). Values are expressed as (%) summing to 100 % for each PC. The overall percentage variation each PC explains is also included in brackets. Only contributions > 1 % are included.

	PC1(68.8 %)	PC2 (18.5 %)	PC3 (8.2 %)
Gram + bacteria	16.7	2.7	3.9
Gram - bacteria	10.4	19.8	9.9
Actinomycetes	13.7	7.5	15.3
SF Fungi	14.2	6.4	6.6
AM Fungi	15.8	5.3	0.5
G+:G-	2.5	46.5	19.6
F:B	9.0	11.4	44.1
Total Biomass	17.7	NA	NA

Table S5 Variable contributions to foliar trait principal components (PC1, PC2 and PC3). Values are expressed as (%) summing to 100 % for each PC. The overall percentage variation each PC explains is also included in brackets. Only contributions > 1 % are included.

	PC1(41%)	PC2 (16.9%)	PC3 (11.8%)
N	11	3.6	2.9
C	5.6	9.6	4.4
C:N	10.7	2.9	2.7
L	1.8	23.8	NA
Cellulose	NA	NA	17
K	10.5	3.1	NA
P	8	3	9.5
Ca	5.8	NA	11.4
Mg	5.9	2.1	15.5
S	11.5	NA	NA
Mn	NA	13.7	15.2
Fe	4.4	8.8	NA
Cu	7.4	11.1	NA
B	3	6.4	5.9
Zn	NA	5.7	13.6
SLA	10.6	NA	NA
Chl	2.2	4	NA

Table S6 Variable contributions to soil properties principal components (PC1, PC2 and PC3). Values are expressed as (%) summing to 100 % for each PC. The overall percentage variation each PC explains is also included in brackets. Only contributions > 1 % are included.

	PC1(67.1%)	PC2 (20.6%)	PC3 (7.7%)
pH	12.3	39.7	43.8
C	27.7	4.9	NA
C:N	14.0	31.9	51.8
Moisture	21.2	17.1	NA
N	24.6	6.5	3.7

Table S7 Variable contributions to bacterial phylum community principal components (PC1, PC2 and PC3). Values are expressed as (%) summing to 100 % for each PC. The overall percentage variation each PC explains is also included in brackets. Only contributions > 1 % are included.

	PC1(62.3%)	PC2 (24.7%)	PC3 (12.9%)
Acidobacteria	31.9	NA	39.4
Actinobacteria	29.5	26.5	NA
Proteobacteria	27.6	NA	59.8
Other	11.0	73.4	NA

Table S8 p-values arising from variation partition modelling in figure 4. See Table S3 for a list of the attributes within each predictor group variable (i.e. Foliar, Soil, Climate, Land cover). Only values with a significance of $p < 0.05$ are shown. All full models are significant ($p < 0.001$).

Microbial Composition	n	Foliar	Soil	Climate	Land cover
Gram + bacteria	96	0.001	0.001	0.03	0.001
Gram - bacteria	96	0.001	0.001	0.001	NA
Actinomycetes	96	0.001	0.001	0.001	0.002
SF Fungi	96	0.001	0.001	NA	0.016
AM Fungi	96	0.001	0.001	0.04	0.008
G+:G-	96	0.001	0.027	0.043	0.001
F:B	96	0.001	0.001	0.001	NA
Total Biomass	96	0.001	0.001	NA	0.004
Acidobacteria	53	NA	NA	NA	NA
Actinobacteria	53	0.045	0.001	NA	NA
Proteobacteria	53	0.031	0.001	0.012	NA
NMDS axis 1	53	NA	0.001	NA	NA
NMDS axis 2	53	0.001	NA	0.001	NA
Bacterial Richness	53	0.001	0.005	NA	NA
Bacterial ASV community matrix	53	0.001	0.003	0.008	NA

Table S9 Summary statistics of PLSR (Partial Least Squares Regression) models built using image-derived plot spectra and plot-level measures of soil microbial community composition. Statistics were generated by comparing the mean predictions of 200 models with unseen validation plots (n = 66 and 65 for the PLFA microbial group and 16S bacterial models; respectively). All models are statistically significant ($p < 0.001$).

	R^2	RMSE	NRMSE (%)	n components
Broad microbial groups (PLFA)				
Gram-positive bacteria (G+)	0.86	18.17	10.9	14
Gram-negative bacteria (G-)	0.61	10.51	14.2	12
Actinomycetales	0.81	9.57	10.1	11
AM fungi	0.86	3.87	10.6	12
SF fungi	0.68	25.14	10.5	12
Total biomass	0.73	68.91	13.8	14
G+:G-	0.73	0.86	12.9	13
F:B	0.67	0.1	11.9	11
Bacterial communities (16S rRNA)				
Acidobacteria	0.29	0.05	19.1	4
Actinobacteria	0.27	0.08	24	4
Proteobacteria	0.3	0.05	22.4	9
Bacterial Richness	0.43	102.83	18.6	6
Bacterial NMDS1	0.61	0.21	15.5	6
Bacterial NMDS 2	0.60	0.13	12.5	6

References

Averill C, Werbin ZR, Atherton KF, Bhatnagar JM, Dietze MC. 2021. Soil microbiome predictability increases with spatial and taxonomic scale. *Nature Ecology & Evolution* **5**(6): 747-+.

Denwood MJ. 2016. runjags: An R Package Providing Interface Utilities, Model Templates, Parallel Computing Methods and Additional Distributions for MCMC Models in JAGS. *Journal of statistical software* **71**(9): 1 - 25.

Pawlowsky-Glahn V, Juan J, Tolosana-Delgado R. 2015. *Modelling and Analysis of Compositional Data*: Wiley.

Plummer M. 2003. JAGS: A program for analysis of Bayesian graphical models using Gibbs sampling. *Proceedings of the 3rd international workshop on distributed statistical computing* 1-10.

Stanish, L. 2024. TOS protocol and procedure: SLS – Soil biogeochemical and microbial sampling. NEON.DOC.014048vP. NEON (National Ecological Observatory Network) https://data.neonscience.org/data-products/DP1.10104.001/RELEASE-2022

Weintraub-Leff, S. 2025. TOS protocol and procedure: CFC – Canopy foliage sampling. NEON.DOC.001024vL. NEON (National Ecological Observatory Network) https://data.neonscience.org/data-products/DP1.10026.001/RELEASE-2022.
